# Neuroprotective Effects of Time‐Restricted Feeding Combined With Different Protein Sources in MPTP‐Induced Parkinson's Disease Mice Model and Its Modulatory Impact on Gut Microbiota Metabolism

**DOI:** 10.1002/advs.202516502

**Published:** 2026-01-26

**Authors:** Ting Li, Jian Wu, Sheng‐Yang Zhou, Ming‐An Li, Li‐Ping Zhao, Ao Wang, Yi‐Zhi Song, Wen‐Yan Huang, Lu‐Lu Tan, Chen‐Meng Qiao, Wei‐Jiang Zhao, Chun Cui, Yan‐Qin Shen

**Affiliations:** ^1^ Laboratory of Neurodegenerative Disease School of Medicine Jiangnan University Wuxi Jiangsu China; ^2^ MOE Medical Basic Research Innovation Center for Gut Microbiota and Chronic Diseases School of Medicine Jiangnan University Wuxi Jiangsu China; ^3^ Affiliated Mental Health Centre of Jiangnan University Wuxi Central Rehabilitation Hospital Wuxi Jiangsu China

**Keywords:** branched‐chain amino acids, casein, gut microbiota, Parkinson's disease, soy protein, time‐restricted feeding

## Abstract

Dietary interventions alleviate Parkinson's disease (PD) progression by modulating the gut microbiota. However, the interaction between time‐restricted feeding (TRF) and dietary protein composition remains unclear. This study examines the effects of casein (animal‐derived) and soy protein (plant‐derived) on PD pathology in an MPTP‐induced mouse model and their influence on TRF efficacy. MPTP induces dopaminergic neuron loss and neuroinflammation regardless of protein source, but casein‐fed mice show partial motor dysfunction and gut barrier disruption, whereas soy protein‐fed mice maintain motor function and barrier integrity. TRF differentially modulates PD outcomes: in casein‐fed mice, it alleviates partial motor deficits by suppressing monoamine oxidase B (MAO‐B) and reducing dopamine (DA) metabolism, without rescuing DA levels or neuron survival. In soy protein‐fed mice, TRF suppresses MAO‐B, preserves dopaminergic neurons, restores DA levels, and reduces neuroinflammation. Analysis of gut microbiota and metabolomics suggests that TRF may reduce *Allobaculum* and branched‐chain amino acids (BCAAs) in casein‐fed mice, while increasing *Akkermansia* and short‐chain fatty acids (SCFAs) in soy protein‐fed mice. Mechanistic assays suggest that *Allobaculum* and BCAAs impair gut barrier function and aggravate inflammation. In conclusion, TRF exerts protein‐dependent neuroprotective effects, with soy protein combined with TRF offering a promising dietary strategy for PD management.

## Graphical abstract

Time‐restricted feeding (TRF) exerts protein‐dependent neuroprotective effects in an MPTP‐induced Parkinson's disease model. In casein‐fed mice, TRF improves gut barrier integrity and reduces neuroinflammation, possibly via modulation of *Allobaculum* and BCAAs. In soy protein‐fed mice, TRF not only attenuates neuroinflammation but also enhances dopaminergic neuron survival, potentially through increased *Akkermansia* abundance and SCFAs. （The graphical abstract was created using MedPeer）

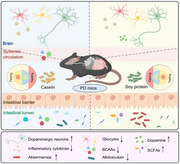

## 1 Introduction

Parkinson's disease (PD), the second most common neurodegenerative disease, is characterized by dopaminergic neuronal degeneration in the substantia nigra (SN), leading to dopamine (DA) depletion in the striatum and subsequent motor impairments. Currently, PD affects approximately 2%–3% of the population over 65 years old, with the number of cases projected to rise from 6.9 million in 2015 to 14.2 million by 2040 [1], making it a growing global concern [2]. Although approximately 5%–10% of PD cases are attributed to genetic factors, the vast majority are believed to result from environmental influences‐among which diet has emerged as a key modulator of disease onset and progression [3]. Notably, accumulating evidence suggests that dietary interventions may exert beneficial effects by shaping the gut microbiota, offering a promising strategy to alleviate PD symptoms and disease progression [4].

In this context, time‐restricted feeding (TRF), a dietary regimen that confines food intake to a specific daily window (4‐ to 12‐h) without restricting caloric intake, has gained widespread attention. TRF has been widely studied in metabolic diseases, demonstrating benefits such as reduced inflammation and modulation of the gut microbiota, both of which are closely associated with PD [5]. Despite these potential benefits, research on the role of TRF in PD remains limited. Moreover, existing studies have predominantly focused on the timing of food intake while largely overlooking the impact of dietary composition, which may critically influence the efficacy of TRF.

Among various dietary components, protein plays a crucial role in maintaining physiological functions and overall health. Importantly, not only the quantity but also the source of protein‐animal‐based versus plant‐based‐can influence health outcome. Epidemiological studies have shown that higher intake of plant‐based proteins is associated with reduced risks of metabolic disorders, including nonalcoholic fatty liver disease, coronary heart disease, and cardiovascular mortality [6, 7, 8]. Notably, plant protein consumption has been shown to attenuate systemic inflammation and modulate gut microbiota composition [9], both of which are closely implicated in gut microbiota dysbiosis and neuroinflammation involved in PD pathogenesis. However, whether combining TRF with different protein sources offers additive or synergistic benefits in the context of PD remains unexplored.

Based on this background, this study aims to evaluate the effects of TRF combined with different protein sources‐casein (animal‐based) and soy protein (plant‐based)‐on PD pathology, neuroinflammation, gut microbiota, and metabolic alterations in MPTP‐induced PD mice. Our findings demonstrate soy protein exerts greater neuroprotective effects than casein and that its combination with TRF further enhances benefits for PD mice. By elucidating the interactions between dietary timing and protein composition, our findings provide an effective dietary strategy for alleviating the progression of PD.

## 2 Results

### 2.1 MPTP‐Induced PD‐Like Pathology Depends on the Source of Dietary Protein

**FIGURE 1:**
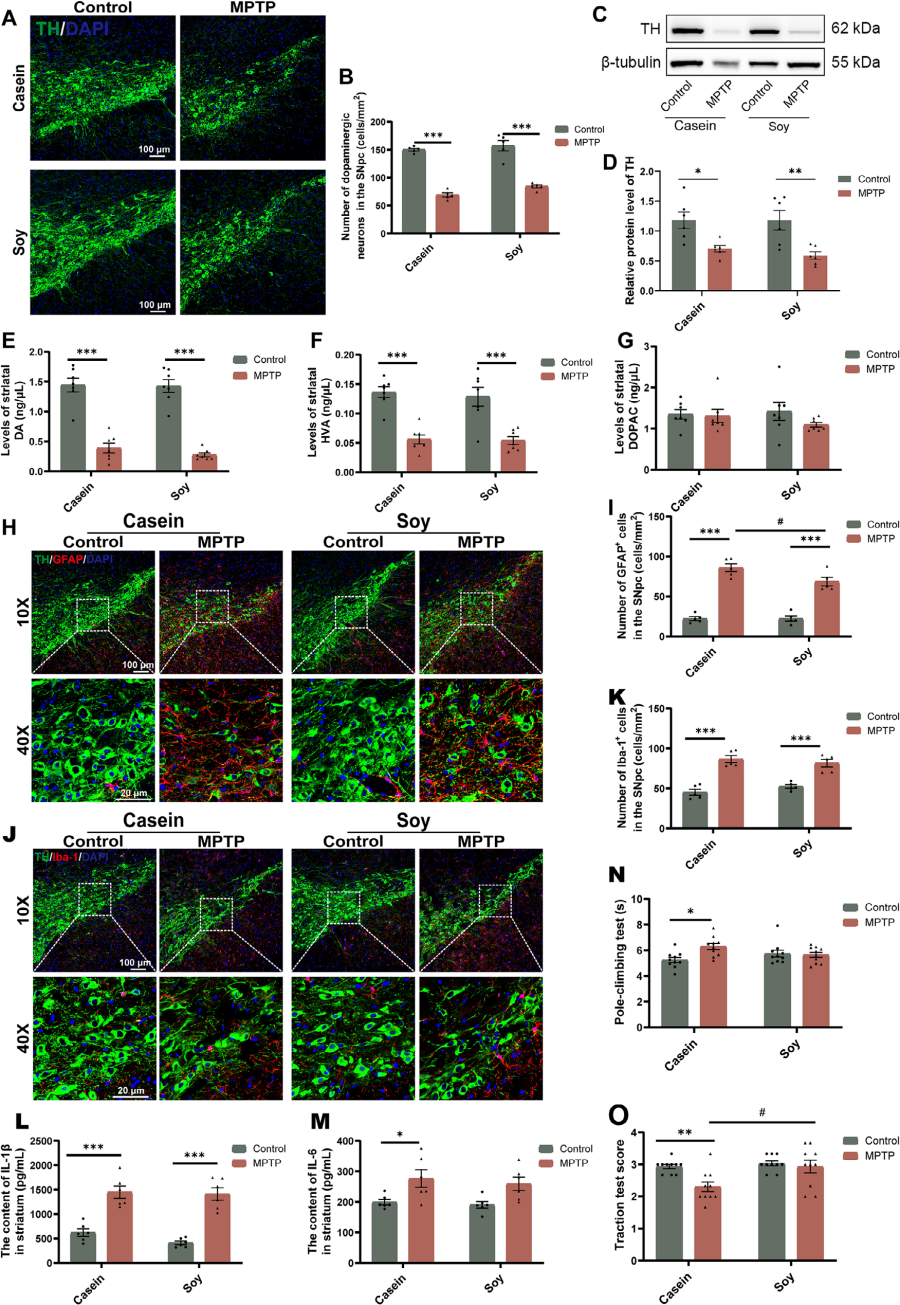
MPTP‐induced PD‐like pathology depends on the source of dietary protein. (A) Representative IF image of TH (green) in the SNpc. Scale bar:100 µm (10×), 20 µm (40×). (B) Quantification of TH‐positive neuronal cells in the SNpc (cells/mm^2^) (n = 5). (C) Representative western blotting of TH protein in the striatum. (D) Quantification of TH protein expression in the striatum (n = 6). (E–G) The concentration of striatal neurotransmitters and metabolites, including DA (E), HVA (F), and DOPAC (G) (n = 7). (H) Double IF staining for TH (dopaminergic neuron marker; green) and GFAP (astrocyte marker; red) in the SNpc. Scale bar:100 µm (10×), 20 µm (40×). (I) Quantitative analysis of the number of activated astrocytes in SNpc (cells mm^−2^) (n = 5). (J) Double IF staining for TH and Iba‐1 (microglial marker; red) in the SNpc. Scale bar:100 µm (10×), 20 µm (40×). (K) Quantitative analysis of the number of activated microglia in SNpc (cells mm^−2^) (n = 5). (L) The protein level of IL‐1β in the striatum (n = 6). (M) The protein level of IL‐6 in the striatum (n = 6). (N) Pole test (n = 10). (O) Traction test (n = 10). Error bars represent the mean ± SEM. Statistical significance for multiple group comparisons was determined using two‐way ANOVA followed by Tukey's post hoc test. Asterisks (*) indicate significant differences among different treatment groups within the same diet (*p < 0.05, **p < 0.01, *p < 0.001), while hash symbols (#) indicate significant differences among treatment groups across different diets (#*p* < 0.05, ##*p* < 0.01, ###*p* < 0.001).

The hallmark pathological features of PD include the degeneration and loss of dopaminergic neurons in the SN, accompanied by a decline in Tyrosine Hydroxylase (TH) expression [10]. Immunofluorescence (IF) and western blotting confirmed that MPTP induced hallmark PD‐related pathology in both casein‐ and soy protein‐fed mice, characterized by a reduction in dopaminergic neurons in the substantia nigra pars compacta (SNpc) (Figure 1A,B), decreased TH levels in the striatum (Figure 1C,D), and a decline in striatal DA and its metabolite Homovanillic acid (HVA), and an increased DA turnover rate (Figure 1E,F, Figure S1B–D). Notably, no significant differences in these key pathological features were observed between the casein‐ and soy protein‐fed PD mice. These findings indicate that MPTP treatment induces dopaminergic neuronal damage, the source of dietary protein does not significantly alter this effect.

MPTP‐induced dopaminergic neuron degeneration is consistently associated with glial activation [11]. MPTP treatment significantly increased the number of glial fibrillary acidic protein (GFAP)‐labeled astrocytes and ionized calcium‐binding adaptor molecule 1 (Iba‐1)‐labeled microglial cells in the SNpc (Figure 1H–K) and striatum (Figure S1E–H), along with elevated levels of the inflammatory cytokine IL‐1β in the striatum (Figure 1L). MPTP treatment also induced an increase in IL‐6 expression in casein‐fed mice (Figure 1M). Notably, casein‐fed PD mice exhibited more severe neuroinflammation than soy protein‐fed PD mice, as evidenced by significantly increased GFAP‐positive astrocyte activation in the SNpc (Figure 1H,I) and striatum (Figure S1E,F), alongside lower mRNA levels of the anti‐inflammatory cytokines *Il‐4* and *Il‐10* (Figure S1I,J).

Behavioral assessments showed that MPTP treatment caused partial motor impairments in mice on a casein diet, as indicated by an increased pole climbing time and lower traction test scores (Figure 1N,O). In contrast, such impairments were not observed in soy protein‐fed PD mice, suggesting that soy protein may have a protective effect against MPTP‐induced partial motor deficits.

To exclude the possibility that the differential PD‐related phenotypes were influenced by diet‐induced systemic metabolic changes, we additionally quantified key metabolic indicators, including glucose, triglycerides, and free fatty acids, in both casein‐ and soy‐fed mice. No significant differences were detected between the two dietary groups (Figure S1K–M). These findings suggest that the observed variations in MPTP‐induced pathology are unlikely to result from alterations in systemic metabolic status, but are instead more likely attributable to differences in amino acid composition and the associated metabolic or microbiota‐mediated effects of each protein source.

### 2.2 MPTP Induces Intestinal Barrier Impairment in Animal‐Derived Casein‐Fed Mice

**FIGURE 2:**
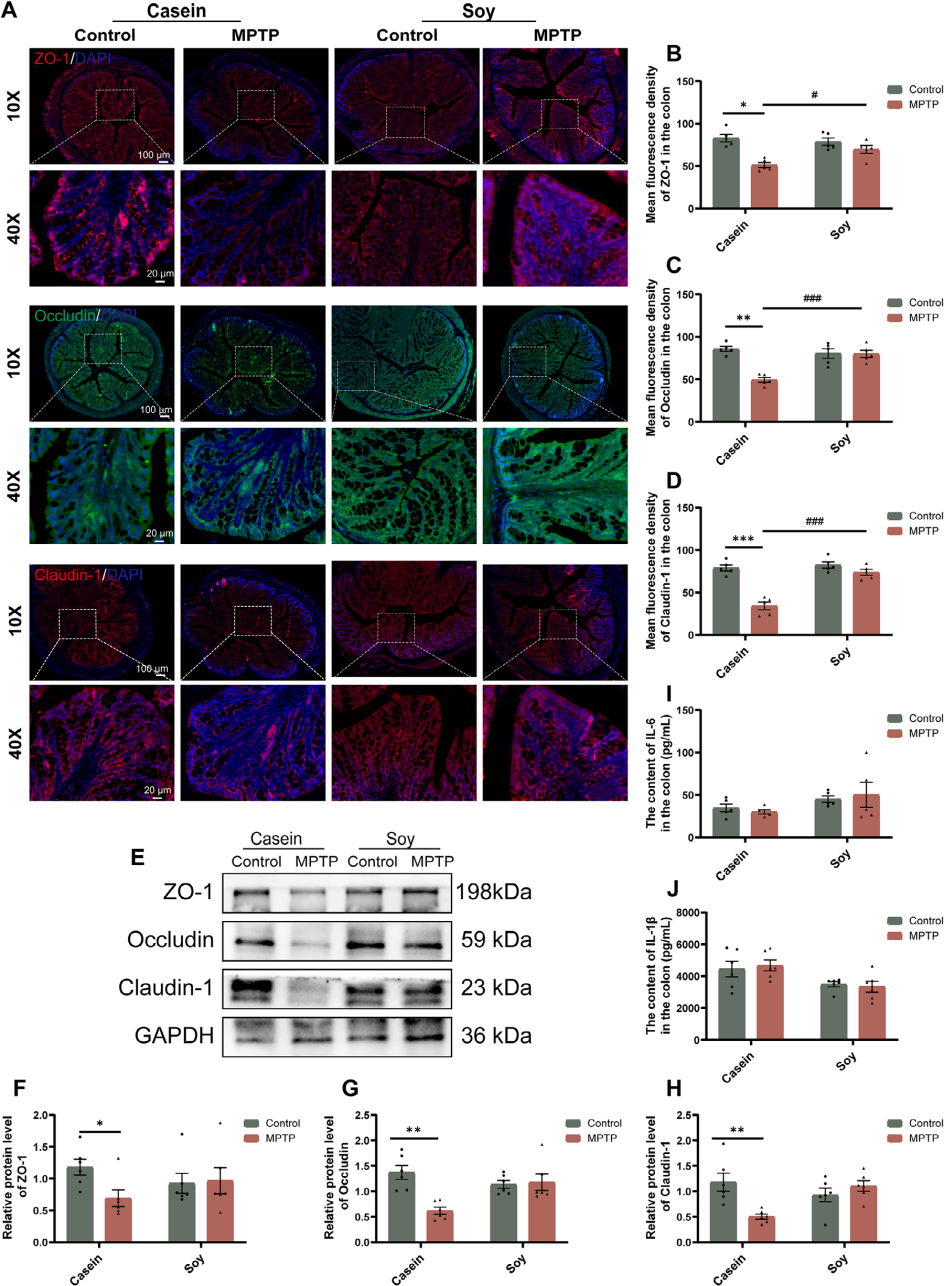
MPTP induces intestinal barrier impairment but not inflammation in animal‐derived casein‐fed mice. (A) Representative IF images of ZO‐1 (red), Occludin (green) and Claudin‐1 (red) in the colon. Scale bar:100 µm (10×), 20 µm (40×). (B–D) Quantification of the mean fluorescence intensity of ZO‐1, Occludin and Claudin‐1 in the colon (n = 5). (E) Representative western blotting of ZO‐1, Occludin and Claudin‐1 proteins in the colon. (F–H) Quantification of ZO‐1, Occludin and Claudin‐1 protein expression in the colon (n = 6). (I) The protein level of IL‐6 in the colon (n = 5). (J) The protein level of IL‐1β in the colon (n = 6). Error bars represent the mean ± SEM. Statistical significance for multiple group comparisons was determined using two‐way ANOVA followed by Tukey's post hoc test. Asterisks (*) indicate significant differences among different treatment groups within the same diet (*p < 0.05, **p < 0.01, *p < 0.001), while hash symbols (#) indicate significant differences among treatment groups across different diets (#*p* < 0.05, ##*p* < 0.01, ###*p* < 0.001).

The intestinal barrier is crucial in PD pathogenesis by preventing bacterial invasion and physical damage [12]. In this study, IF staining showed that casein‐fed PD mice exhibited a significant reduction in the mean fluorescence intensity of tight junction proteins (ZO‐1, Occludin, and Claudin‐1) in the colon, whereas this reduction was not observed in soy protein‐fed PD mice (Figure 2A–D). This result was further confirmed by western blotting, which showed that casein‐fed PD mice markedly decreased the protein expression of ZO‐1, Occludin and Claudin‐1 in the colon, but not soy protein‐fed mice (Figure 2E–H). These findings suggest that dietary soy protein may exert a protective effect against MPTP‐induced intestinal barrier disruption. Notably, despite MPTP‐induced intestinal barrier impairment, no significant increase in colonic inflammatory cytokines (IL‐6 and IL‐1β) was observed in either dietary group (Figure 2I,J), indicating that intestinal barrier dysfunction occurred independently of colonic inflammation in this context.

### 2.3 MPTP‐Induced Gut Microbiota Alterations Depend on the Source of Dietary Protein

**FIGURE 3:**
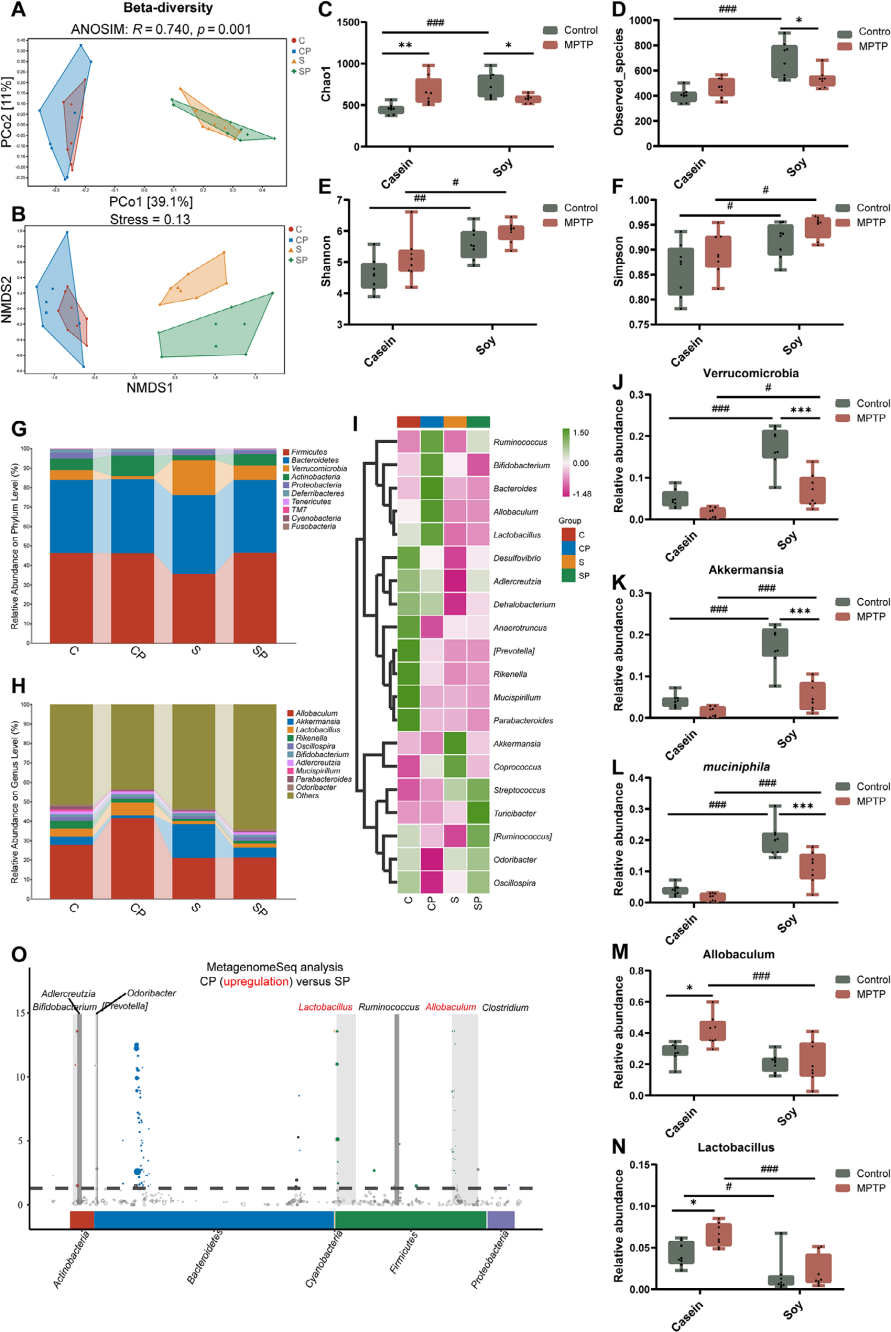
Effects of animal‐derived casein and plant‐derived soy protein on gut microbiota in the MPTP‐induced PD model. (A) Principal coordinate analysis (PCoA) plot of β‐diversity based on Bray‐Curtis analysis of similarities (ANOSIM) analysis using the relative abundance of OTUs (n = 8). (B) Nonmetric multidimensional scaling (NMDS) analysis of β‐diversity based on Bray‐Curtis ANOSIM analysis using the relative abundance of OTUs (n = 8). (C–F) α‐diversity indices of the gut microbiota (n = 8). (G) Relative abundance distribution of the top 10 most abundant gut microbiota at the phylum level (n = 8). (H) Relative abundance distribution of the top 10 most abundant gut microbiota at the genus level (n = 8). (I) Heatmap analysis of the relative abundance of the top 20 most abundant gut microbiota at the genus level (n = 8). (J) Relative abundance of Verrucomicrobia (n = 8). (K) Relative abundance of *Akkermansia* (n = 8). (L) Relative abundance of *muciniphila* (n = 8). (M) Relative abundance of *Allobaculum* (n = 8). (N) Relative abundance of *Lactobacillus* (n = 8). (O) Genus with significant difference between CP and SP groups in MetagenomeSeq analysis. C: casein‐fed mice, CP: casein‐fed PD mice, S: soy protein‐fed mice, SP: soy protein‐fed PD mice. Error bars represent the mean ± SEM. Statistical significance for multiple group comparisons was determined using two‐way ANOVA followed by Tukey's post hoc test. Asterisks (*) indicate significant differences among different treatment groups within the same diet (*p < 0.05, **p < 0.01, *p < 0.001), while hash symbols (#) indicate significant differences among treatment groups across different diets (#*p* < 0.05, ##*p* < 0.01, ###*p* < 0.001).

To investigate whether the differential effects of MPTP treatment depend on dietary protein source and are associated with gut microbiota alterations, we performed 16S rRNA sequencing to analyze the microbiota composition and infer potential functional changes. β‐diversity analysis revealed distinct differences in microbial community composition between the casein‐ and soy protein‐fed groups (Figure 3A,B). Additionally, α‐diversity analysis indicated that casein‐fed PD mice increased microbial richness (Chao1 and Observed‐species indices) but decreased it in soy protein‐fed PD mice (Figure 3C,D). Notably, despite a reduction in microbial richness, soy protein‐fed PD mice still exhibited higher overall microbial diversity (Simpson and Shannon indices) than casein‐fed PD mice (Figure 3E,F). These findings suggest that dietary protein sources modulate MPTP‐induced gut microbiota alterations, with plant‐derived soy protein potentially promoting greater microbial diversity even under PD‐related conditions. Further taxonomic analysis revealed that, at the phylum and genus levels, MPTP treatment induced distinct microbial changes between the two dietary groups (Figure 3G–I). In soy protein‐fed mice, MPTP treatment significantly reduced the relative abundance of Verrucomicrobia (Figure 3J) and its representative species, *Akkermansia* and *muciniphila* (Figure 3K,L). Interestingly, despite this reduction, soy protein‐fed PD mice still retained a higher abundance of *Akkermansia* and *muciniphila* compared to casein‐fed PD mice (Figure 3K,L). *Akkermansia muciniphila* (*A. muciniphila*) has been reported to enhance gut barrier function and promote anti‐inflammatory immune responses [13], which may help explain the absence of gut barrier dysfunction and intestinal inflammation observed in soy protein‐fed PD mice (Figure 2). In casein‐fed mice, MPTP significantly increased the abundance of *Allobaculum* and *Lactobacillus* (Figure 3M,N). Casein‐fed PD mice exhibited a higher abundance of *Lactobacillus* compared to those on a soy protein diet (Figure 3N). Notably, *Lactobacillus* has potent anti‐inflammatory effects [14, 15], and its increased abundance in casein‐fed PD mice may help explain the absence of intestinal inflammation, despite the presence of intestinal barrier disruption. Further analysis using MetagenomeSeq confirmed that *Allobaculum* and *Lactobacillus* were the dominant taxa in casein‐fed PD mice (Figure 3O). Taken together, these findings suggest that dietary protein sources modulate MPTP‐induced changes in gut microbiota, which may partly contribute to the distinct intestinal pathologies observed in PD mice.

### 2.4 TRF Modulates Dopaminergic Neurons and Motor Function in PD Mice Depending on Dietary Protein Source

**FIGURE 4:**
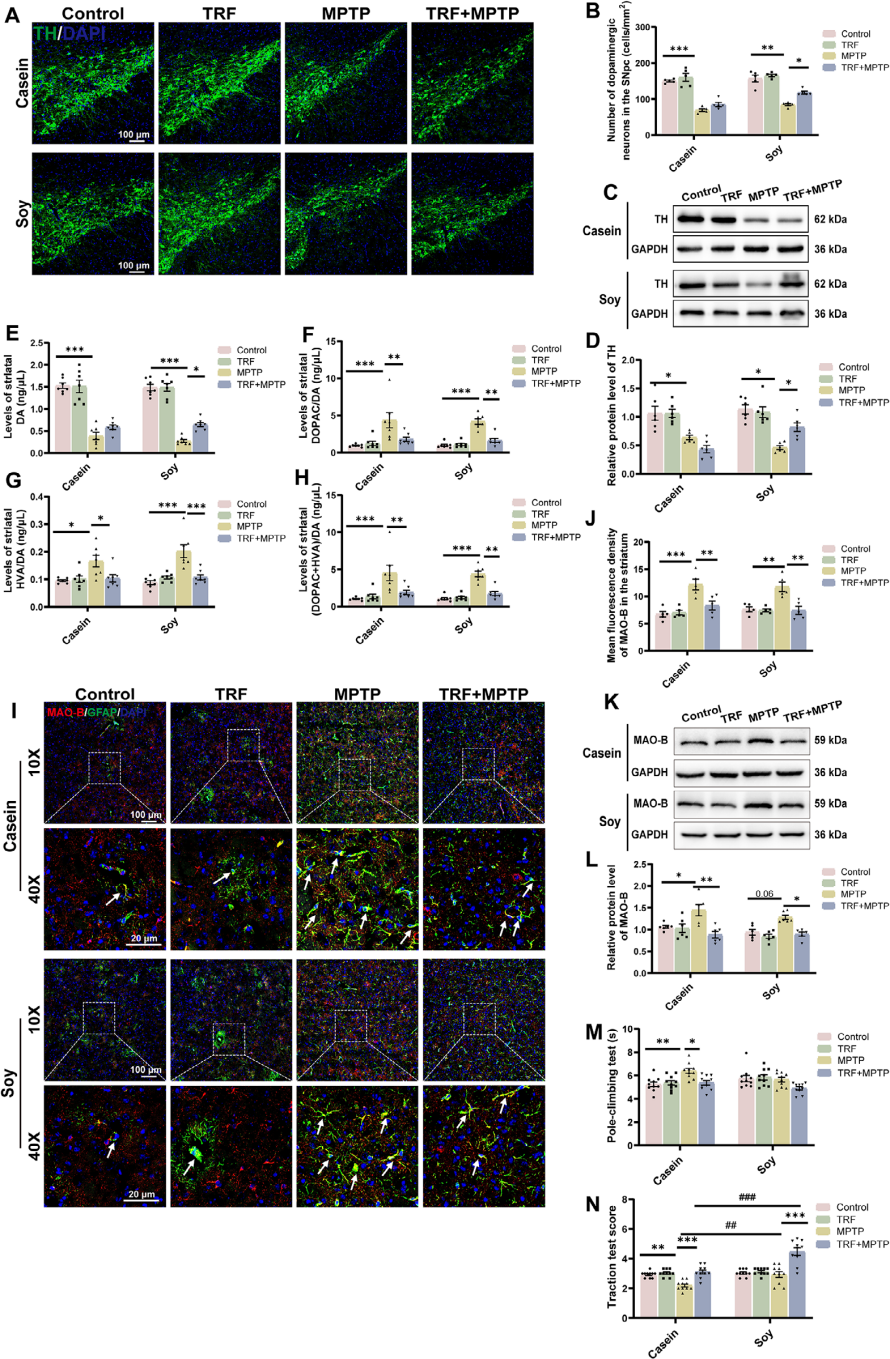
TRF modulates dopaminergic neurons and motor function in PD mice depending on dietary protein source. (A) Representative IF of TH (green) in the SNpc. Scale bar: 100 µm (10×), 20 µm (40×). (B) Quantification of TH‐positive neurons in the SNpc (cells/mm^2^) (n = 5). (C) Representative western blotting of TH protein in the striatum. (D) Quantification of TH protein expression in the striatum (n = 6). (E–H) The concentration of striatal neurotransmitters and metabolic rate, including DA (E), ratio of DOPAC/DA (F), ratio of HVA/DA (G), and ratio of (DOPAC+HVA)/DA (H) (n = 7). (I) Double IF staining for MAO‐B (red) and GFAP (green) in the striatum. Scale bar: 100 µm (10×), 20 µm (40×). (J) Quantification of the mean fluorescence intensity of MAO‐B in the striatum (n = 5). (K) Representative western blotting of MAO‐B protein in the striatum. (L) Quantification of MAO‐B protein expression in the striatum (n = 6). (M) Pole test (n = 10). (N) Traction test (n = 10). Error bars represent the mean ± SEM. Statistical significance for multiple group comparisons was determined using two‐way ANOVA followed by Tukey's post hoc test. Asterisks (*) indicate significant differences among different treatment groups within the same diet (*p < 0.05, **p < 0.01, *p < 0.001), while hash symbols (#) indicate significant differences among treatment groups across different diets (#*p* < 0.05, ##*p* < 0.01, ###*p* < 0.001).

MPTP induces motor dysfunction and intestinal barrier impairment in casein‐fed mice, whereas a soy protein diet confers protective effects. This raises the question of whether TRF, in combination with different protein sources, can further alleviate MPTP‐induced PD‐like pathology. A four‐week TRF regimen did not alter the number of dopaminergic neurons (Figure 4A–D), striatal DA content (Figure 4E) or its metabolite HVA and 3,4‐Dihydroxyphenylacetic acid (DOPAC) (Figure S2D,E) in casein‐fed PD mice. However, TRF significantly reduced the DA metabolic rate (Figure 4F–H), which was accompanied by a marked decrease in striatal MAO‐B expression (Figure 4I–L). MAO‐B, a key enzyme primarily expressed in astrocytes, plays a crucial role in dopamine metabolism [16]. Despite having no significant impact on dopaminergic neuronal survival, TRF partially alleviated MPTP‐induced motor deficits in casein‐fed PD mice, as evidenced by behavioral assessments (Figure 4M,N). These findings suggest that TRF may partially improve motor symptoms by modulating dopamine metabolism in casein‐fed PD mice. Body weight and food intake were monitored throughout the experiment, and no significant differences were observed among groups, indicating that the observed effects of TRF were independent of caloric intake or body weight (Figure S2B,C).

In contrast, in soy protein‐fed PD mice, TRF not only improved the DA metabolic rate and reduced MAO‐B expression but also significantly increased the number of dopaminergic neurons, TH expression, and DA content in the striatum (Figure 4A–L). Together, these findings suggest that TRF exerts distinct effects on the dopaminergic system in MPTP‐induced PD mice depending on dietary protein sources. The combination of TRF and plant‐derived soy protein further enhances its neuroprotective effects in PD mice.

### 2.5 TRF Suppresses Neuroinflammation in PD Mice Independently of Dietary Protein Source

**FIGURE 5:**
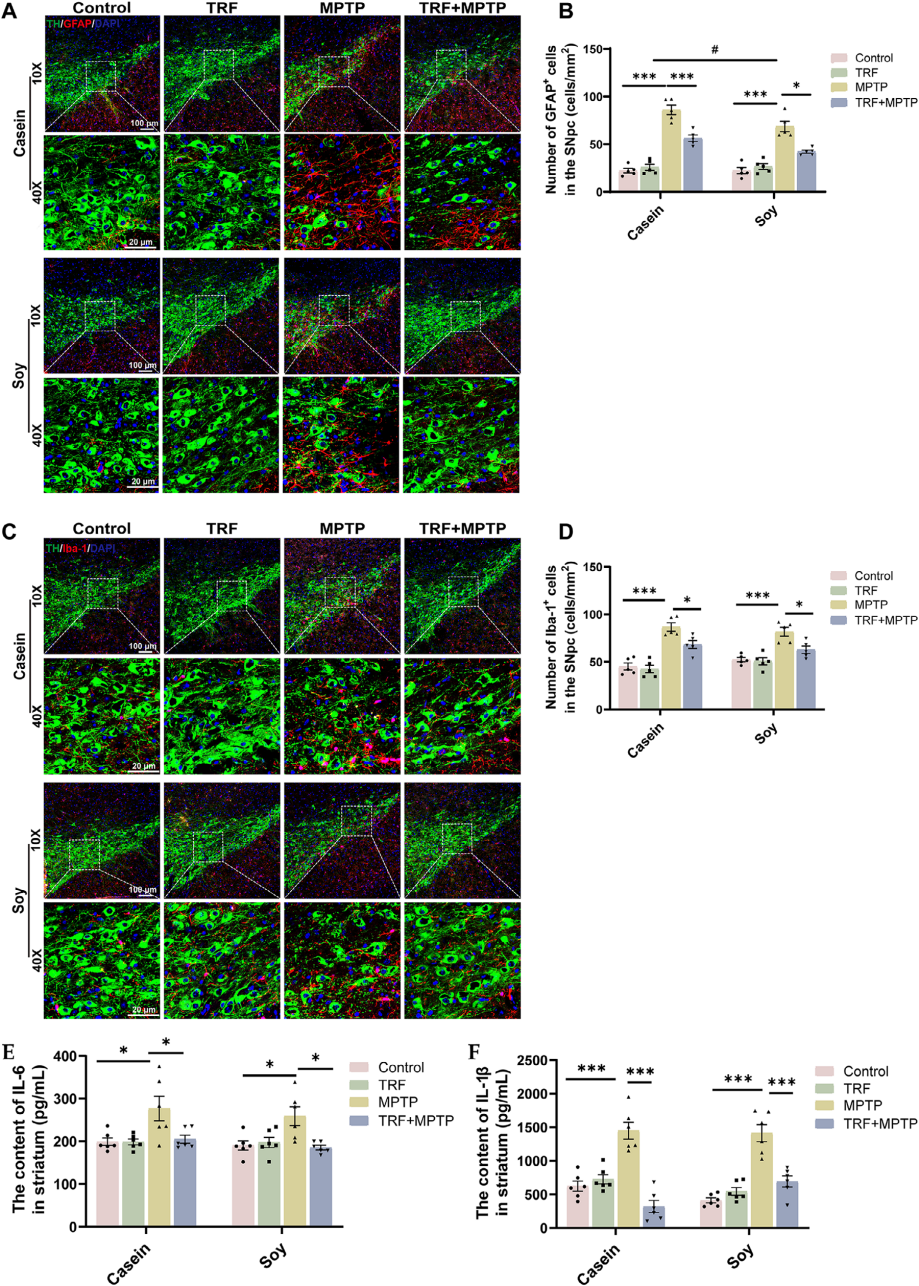
TRF suppresses neuroinflammation in PD mice independently of dietary protein source. (A) Double IF staining for TH (green) and GFAP (red) in the SNpc. Scale bar: 100 µm (10×), 20 µm (40×). (B) Quantification of activated astrocytesin the SNpc (cells mm^−2^) (n = 5). (C) Double IF staining for TH and Iba‐1 (red) in the SNpc. Scale bar: 100 µm (10×), 20 µm (40×). (D) Quantification of activated microglia in the SNpc (cells mm^−2^) (n = 5). (E) Protein level of IL‐6 in the striatum (n = 6). (F) Protein level of IL‐1β in the striatum (n = 6). Error bars represent the mean ± SEM. Statistical significance for multiple group comparisons was determined using two‐way ANOVA followed by Tukey's post hoc test. Asterisks (*) indicate significant differences among different treatment groups within the same diet (*p < 0.05, **p < 0.01, *p < 0.001), while hash symbols (#) indicate significant differences among treatment groups across different diets (#*p* < 0.05, ##*p* < 0.01, ###*p* < 0.001).

IF analysis revealed that TRF significantly attenuated glial activation in the SNpc and striatum of PD mice, as evidenced by reductions in GFAP‐positive astrocytes and Iba‐1‐positive microglia (Figure 5A–D, Figure S2F–I), regardless of the dietary protein source. Glial cells play a pivotal role in PD‐related neuroinflammation by releasing proinflammatory cytokines [17]. Consistently, TRF markedly decreased striatal levels of the proinflammatory cytokines IL‐6 and IL‐1β (Figure 5E,F). These findings suggest that TRF effectively suppresses glial cell activation and neuroinflammation in PD mice, and this effect is independent of dietary protein source since, as casein‐ and soy protein‐fed mice exhibited similar neuroinflammatory responses.

### 2.6 TRF Reverses Gut Barrier Dysfunction in Casein‐Fed PD Mice

**FIGURE 6:**
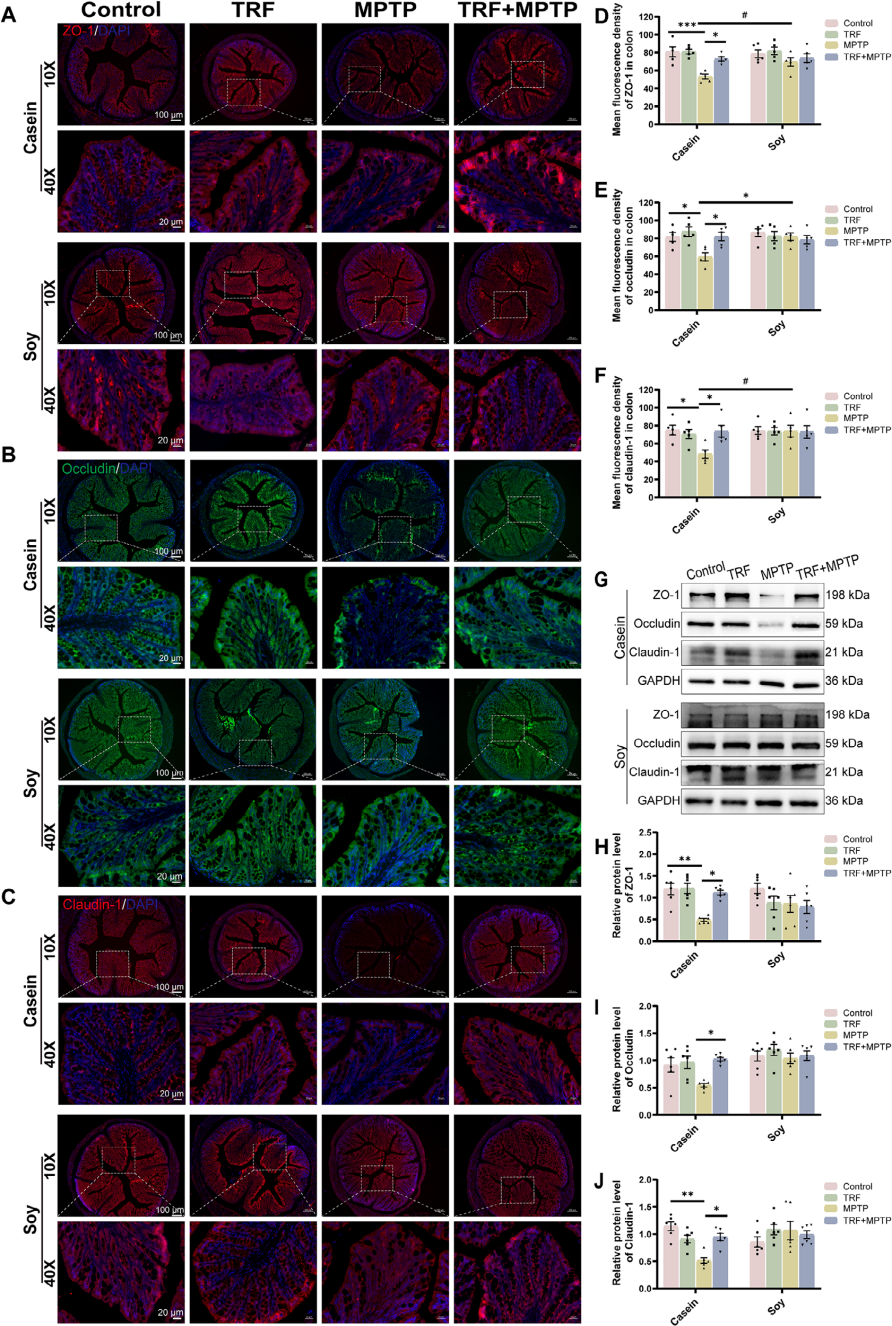
TRF reverses gut barrier dysfunction in casein‐fed PD mice. (A–C) Representative IF images of ZO‐1 (red), Occludin (green), and Claudin‐1 (red) in the colon. Scale bar: 100 µm (10×), 20 µm (40×). (D–F) Quantification of the mean fluorescence intensity of ZO‐1, Occludin, and Claudin‐1 in the colon (n = 5). (G) Representative western blotting of ZO‐1, Occludin, and Claudin‐1 proteins in the colon. (H–J) Quantification of ZO‐1, Occludin, and Claudin‐1 protein expression in the colon (n = 6). Error bars represent the mean ± SEM. Statistical significance for multiple group comparisons was determined using two‐way ANOVA followed by Tukey's post hoc test. Asterisks (*) indicate significant differences among different treatment groups within the same diet (*p < 0.05, **p < 0.01, *p < 0.001), while hash symbols (#) indicate significant differences among treatment groups across different diets (#*p* < 0.05, ##*p* < 0.01, ###*p* < 0.001).

To assess the impact of TRF on MPTP‐induced gut barrier dysfunction in casein‐fed PD mice, IF and western blotting analyses were performed to examine the expression of key tight junction proteins. TRF significantly restored the levels of ZO‐1, Occludin, and Claudin‐1 in casein‐fed PD mice (Figure 6A–J). However, no significant differences were observed in colonic proinflammatory cytokines IL‐6 and IL‐1β among the groups (Figure S2J, K). These findings indicate that TRF effectively promotes gut barrier integrity that was impaired by MPTP induction.

### 2.7 TRF Modulates Gut Microbiota in PD Mice in a Protein Diet‐Dependent Manner in PD Mice

**FIGURE 7:**
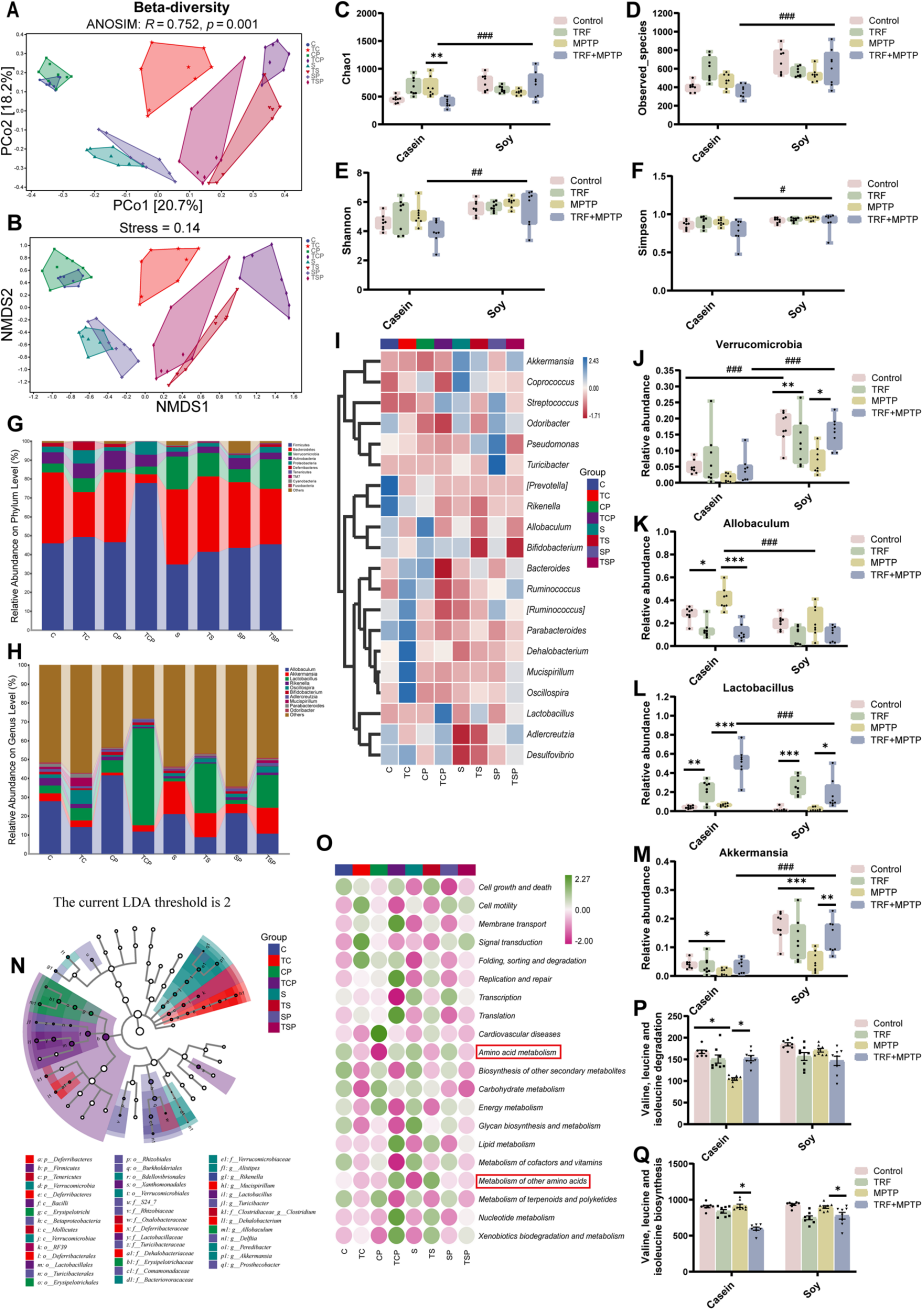
TRF modulates gut microbiota in PD mice in a protein diet‐dependent manner. (A) PCoA plots of β‐diversity based on Bray‐Curtis ANOSIM analysis using the relative abundance of OTUs (n = 8). (B) NMDS analysis of β‐diversity based on Bray‐Curtis ANOSIM analysis using the relative abundance of OTUs (n = 8). (C–F) α‐diversity of gut microbiota (n = 8). (G) Relative abundance distribution of the top 10 most abundant gut microbiota at the phylum level (n = 8). (H) Relative abundance distribution of the top 10 most abundant gut microbiota at the genus level (n = 8). (I) Heatmap analysis of the relative abundance of the top 20 most abundant gut microbiota at the genus level (n = 8). (J) Relative abundance of Verrucomicrobia (n = 8). (K–M) Relative abundance of three significantly altered bacterial genera: *Allobaculum*, *Lactobacillus*, *Akkermansia* (n = 8). (N) Cladogram based on LEfSe analysis of gut microbiota in different groups, using an LDA score threshold of >2.0 and a *p*‐value of <0.05 (n = 8). (O) Predictive metabolism pathways on KEGG level II based on PICRUSt analysis (n = 8). The red frames represented the metabolic pathways related to amino acids in the gut microbiota. (P) The predictive BCAAs degradation pathways on KEGG level III based on PICRUSt analysis (n = 8). (Q) The predictive BCAAs biosynthesis pathways on KEGG level III based on PICRUSt analysis (n = 8). C: casein‐fed mice, TC: TRF‐treated casein‐fed mice, CP: casein‐fed PD mice, TCP: TRF‐treated casein‐fed PD mice; S: soy protein‐fed mice, TS: TRF‐treated soy protein‐fed mice, SP: soy protein‐fed PD mice, TSP: TRF‐treated soy protein‐fed PD mice. Error bars represent the mean ± SEM. Statistical significance for multiple group comparisons was determined using two‐way ANOVA followed by Tukey's post hoc test. Asterisks (*) indicate significant differences among different treatment groups within the same diet (*p < 0.05, **p < 0.01, *p < 0.001), while hash symbols (#) indicate significant differences among treatment groups across different diets (#*p* < 0.05, ##*p* < 0.01, ###*p* < 0.001).

To assess the effects of TRF on gut microbiota composition in PD mice, we conducted β‐ and α‐diversity analyses. β‐diversity analysis revealed that TRF induced distinct alterations in gut microbiota composition in PD mice with different dietary protein sources (Figure 7A,B), whereas α‐diversity analysis showed that TRF did not significantly affect microbial diversity within the same protein diet (Figure 7C–F). However, under different protein conditions, TRF increased microbial richness and diversity in soy protein‐fed PD mice compared to casein‐fed PD mice. These results suggest that the effect of TRF on gut microbiota composition depends on the protein source.

At the phylum level, Firmicutes, Verrucomicrobia, and Bacteroidetes were the dominant taxa (Figure 7G). TRF significantly increased the abundance of Verrucomicrobia in soy protein‐fed PD mice (Figure 7J). At the genus level, *Allobaculum*, *Lactobacillus*, and *Akkermansia* were the dominant taxa (Figure 7H,I). TRF significantly increased *Lactobacillus* and decreased *Allobaculum* in casein‐fed PD mice (Figure 7K,L), while also enhancing *Akkermansia* in soy protein‐fed PD mice (Figure 7M). Notably, TRF‐treated casein‐fed PD mice exhibited a higher abundance of *Lactobacillus*, whereas *Akkermansia* was more abundant in soy protein‐fed PD mice. Linear Discriminant Analysis Effect Size (LEfSe) analysis identified *Allobaculum* as a key bacterial genus in casein‐fed PD mice (Figure 7N), suggesting that it may play an important role under dietary casein conditions.

The composition of the gut microbiota shapes its functional capacity. To explore functional changes, Phylogenetic Investigation of Communities by Reconstruction of Unobserved States (PICRUSt2) was used to infer the relative abundance of Kyoto Encyclopedia of Genes and Genomes (KEGG) pathways. The results revealed that microbial functions were predominantly related to metabolism (Figure S3A). Further analysis of KEGG secondary metabolic pathways indicated potential alterations in amino acid metabolism and metabolism of other amino acids in TRF‐treated mice (Figure 7O). At KEGG level III, the gut microbiota of casein‐fed PD mice exhibited a greater predicted capacity for BCAAs (valine, leucine, and isoleucine) biosynthesis, accompanied by a reduced capacity for their degradation (Figure 7P,Q). Notably, TRF appeared to suppress BCAAs biosynthesis while promoting their degradation. In contrast, such predicted BCAAs metabolic disturbances were not observed in soy protein‐fed PD mice. These results suggest that TRF may modulate the gut microenvironment and overall metabolic potential of casein‐fed PD mice, possibly through regulation of BCAAs metabolism.

### 2.8 TRF Modulates BCAAs Metabolism and SCFAs in a Protein Diet‐Dependent Manner in PD Mice

**FIGURE 8:**
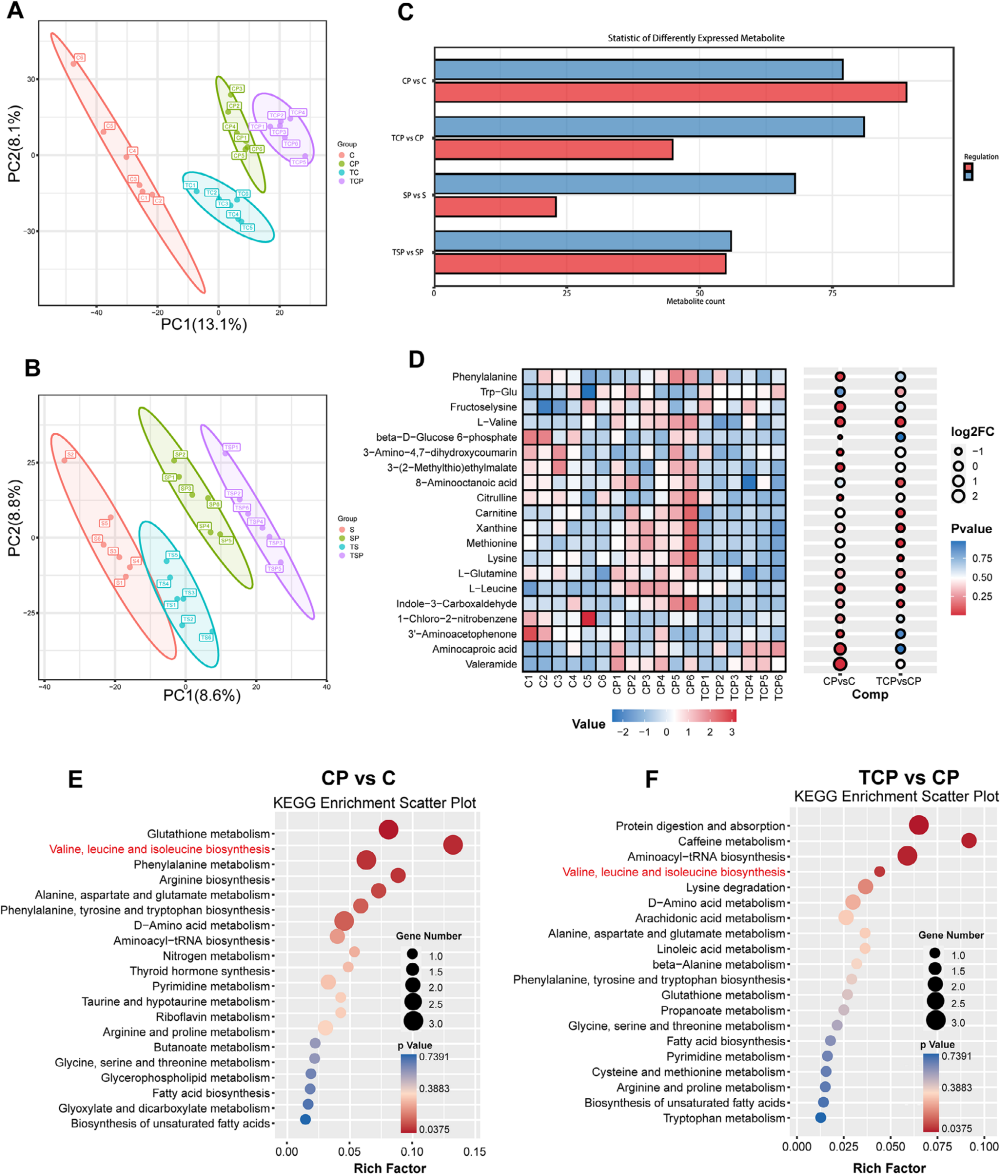
TRF modulates metabolic pathways in a diet‐dependent manner. (A,B) PCA of serum metabolites in casein group (A) and soy protein group (B). (C) The number of differentially expressed metabolites between groups. Red and blue bars indicate upregulated and downregulated metabolites, respectively. (D) Heatmap of representative differentially expressed metabolites with corresponding log2 fold change (log2FC) and p values. (E,F) KEGG pathway enrichment analysis for CP vs C (E) and TCP vs CP (F). Bubble size represents the number of mapped genes, and color indicates p value. Key amino acid metabolism pathways, including valine, leucine, and isoleucine biosynthesis, are highlighted in red. C: casein‐fed control mice; CP: casein‐fed PD mice; TCP: TRF‐treated casein‐fed PD mice; S: soy protein‐fed control mice; SP: soy protein‐fed PD mice; TSP: TRF‐treated soy protein‐fed PD mice.

Predicted functional analysis using PICRUSt2 suggested that TRF may modulate pathological features of casein‐fed PD mice by regulating BCAAs metabolism. To validate these predictions, serum samples were analyzed using untargeted metabolomics. PCA and OPLS‐DA revealed distinct metabolic profiles among groups, with dietary protein source strongly influencing the changes (Figure 8A,B, Figure S3B–E). MPTP induced widespread metabolic alterations under both casein and soy protein diets, whereas TRF partially reversed these effects, including reductions in valine and leucine levels (Figure 8C,D, Figure S3F). KEGG pathway analysis further revealed that, under the casein diet, the most significantly affected pathways were glutathione metabolism, BCAAs (valine, leucine, and isoleucine) biosynthesis, and phenylalanine metabolism. In contrast, under the soy protein diet, enriched pathways primarily involved protein digestion and absorption, suggesting that the effects of TRF on metabolic pathways are influenced by the dietary protein source (Figure S3G,H).

**FIGURE 9:**
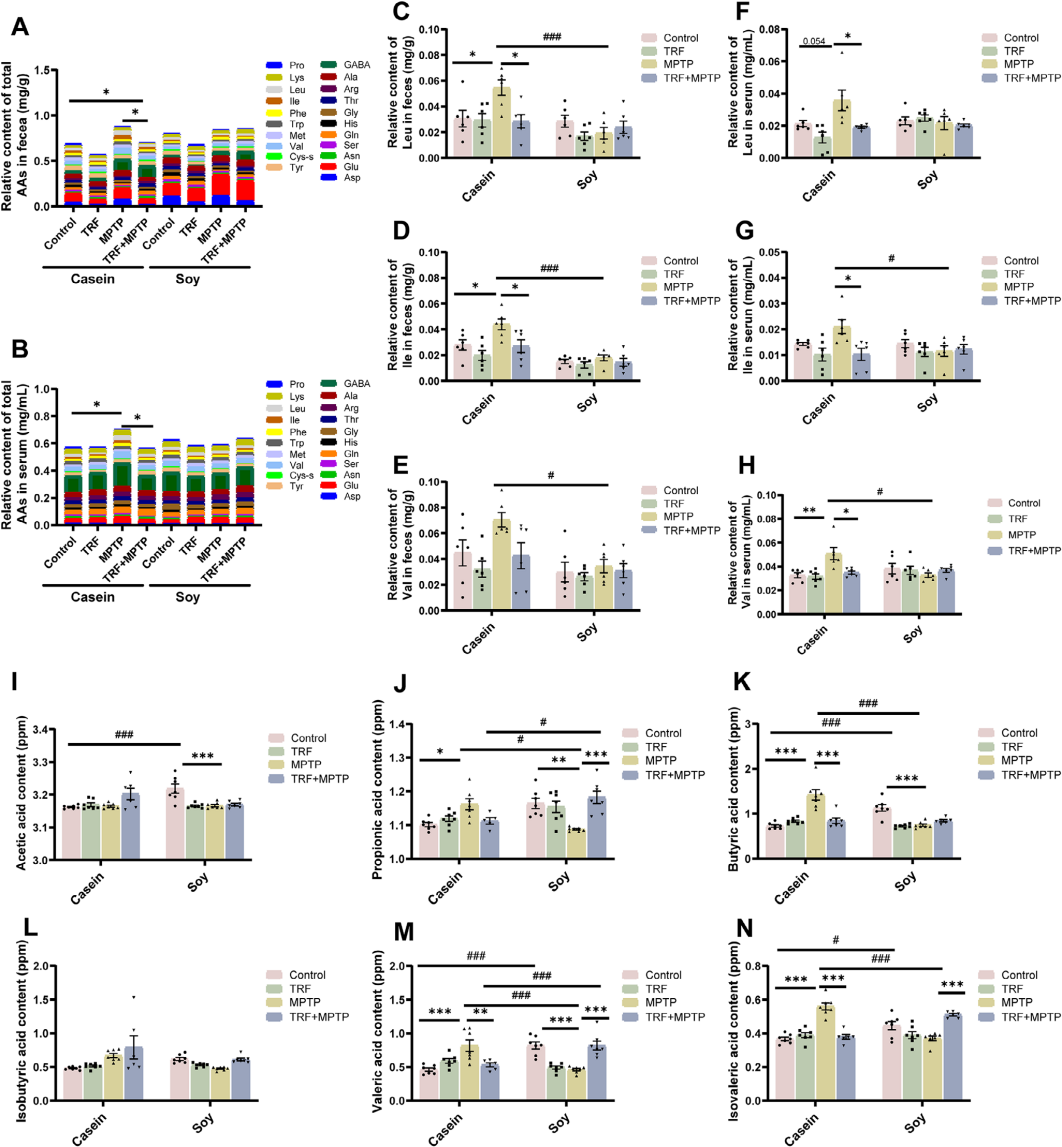
TRF modulates BCAAs metabolism and SCFAs in a protein diet‐dependent manner in PD mice. (A) Total fecal amino acid concentration (n = 6). (B) Total serum amino acid concentration (n = 6). (C–E) Concentration of fecal leucine, isoleucine, and valine (n = 6). (F–H) Concentration of serum leucine, isoleucine and valine (n = 6). (I–N) Concentration of fecal acetic acid, propanoic acid, butyric acid, isobutyric acid, valeric acid, and isovaleric acid (n = 7). Error bars represent the mean ± SEM. Statistical significance for multiple group comparisons was determined using two‐way ANOVA followed by Tukey's post hoc test. Asterisks (*) indicate significant differences among different treatment groups within the same diet (*p < 0.05, **p < 0.01, *p < 0.001), while hash symbols (#) indicate significant differences among treatment groups across different diets (#*p* < 0.05, ##*p* < 0.01, ###*p* < 0.001).

Therefore, to validate the metabolomic findings and further elucidate the relationship between gut microbiota alterations and amino acid metabolism, we measured amino acid concentrations in fecal and serum samples (Figure 9A–H). The results showed that total fecal amino acid levels were significantly higher in casein‐fed PD mice, an effect that was markedly reversed by TRF (Figure 9A). A similar trend was observed in total amino acid concentrations in the blood (Figure 9B), suggesting that TRF modulates systemic BCAAs metabolism in casein‐fed PD mice. Further analysis revealed a significant elevation in leucine and isoleucine levels in both feces and serum of casein‐fed PD mice, which was effectively attenuated by TRF (Figure 9C–H). Meanwhile, TRF increased the BCAAs catabolic enzyme BCAT1 in the colonic tissue of casein‐fed PD mice (Figure S3I). In contrast, no significant differences in total amino acid levels, or BCAAs were observed in the feces or serum of soy protein‐fed mice (Figure 9C–H). These findings suggest that MPTP‐induced alterations in BCAAs metabolism in PD mice depend on dietary protein source, while TRF restores MPTP‐induced BCAAs metabolic disturbances in PD mice.

BCAAs can be metabolized into branched‐chain SCFAs (BSCFAs) through proteolytic fermentation in the gut [18], a special type of SCFAs. Since MPTP induced the accumulation of fecal and serum BCAAs in casein‐fed mice, while TRF enhanced BCAAs degradation, we measured fecal SCFA levels, including BSCFAs such as isobutyric acid and isovaleric acid, in each group. In casein‐fed PD mice, the levels of several SCFAs, including butyric acid, valeric acid, and isovaleric acid, were significantly elevated, which may be associated with gut barrier impairment induced by the casein diet [19, 20], and TRF markedly reduced their concentrations. Interestingly, in soy protein‐fed PD mice, acetic acid, propionic acid, butyric acid, and valeric acid levels were significantly decreased following MPTP treatment, but TRF selectively restored propionic acid and valeric acid levels (Figure 9I–N). These findings indicate that MPTP‐induced alterations in fecal SCFAs in PD mice depend on dietary protein source, even within the same PD model, and that TRF may help restore SCFAs homeostasis in a diet‐dependent manner.

### 2.9 Excessive BCAAs Aggravate Neuroinflammation Through the Akt/STAT3/NF‐κB Pathway in Vitro

**FIGURE 10:**
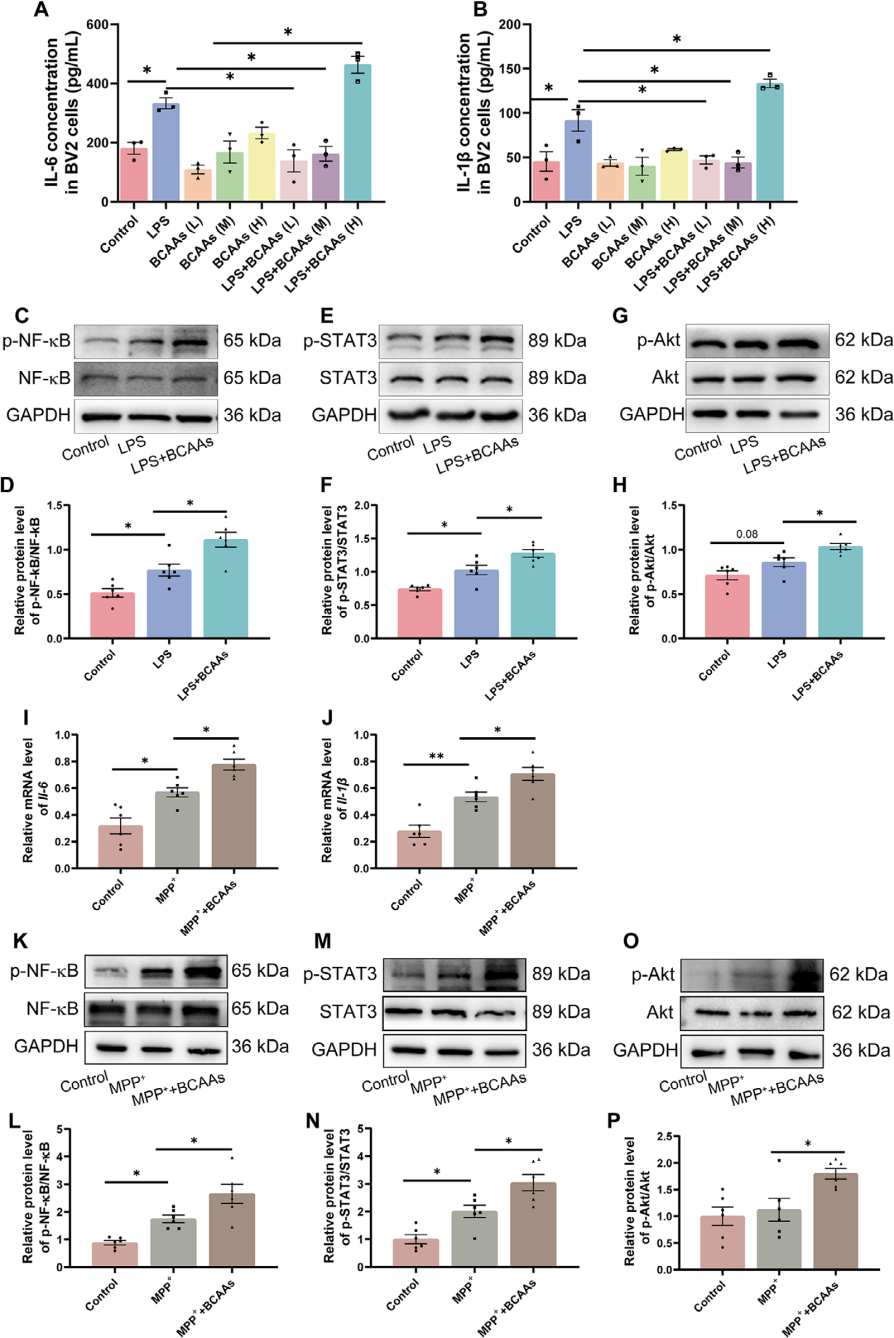
Excessive BCAAs aggravates neuroinflammation through the Akt/STAT3/NF‐κB pathway in vitro. (A) IL‐6 concentration in BV2 cells (pg mL^−1^) (n = 3). (B) IL‐1β concentration in BV2 cells (pg mL^−1^) (n = 3). (C) Representative western blotting of p‐NF‐κB and NF‐κB in BV2 cells. (D) Quantification of p‐NF‐κB/NF‐κB in BV2 cells (n = 6). (E) Representative western blotting of p‐STAT3 and STAT3 in BV2 cells. (F) Quantification of p‐STAT3/STAT3 in BV2 cells (n = 6). (G) Representative western blotting of p‐Akt and Akt in BV2 cells. (H) Quantification of p‐Akt/Akt in t BV2 cells (n = 6). (I) The mRNA level of *Il‐6* in SH‐SY5Y cells (n = 6). (J) The mRNA level of *Il‐1β* in SH‐SY5Y cells (n = 6). (K) Representative western blotting of p‐NF‐κB and NF‐κB in SH‐SY5Y cells. (L) Quantification of p‐NF‐κB/NF‐κB in SH‐SY5Y cells (n = 6). (M) Representative western blotting of p‐STAT3 and STAT3 in SH‐SY5Y cells. (N) Quantification of p‐STAT3/STAT3 in SH‐SY5Y cells (n = 6). (O) Representative western blotting of p‐Akt and Akt in the SH‐SY5Y cell. (P) Quantification of p‐Akt/Akt in the SH‐SY5Y cells (n = 6). Error bars represent the mean ± SEM. Statistical significance for multiple group comparisons was determined using one‐way ANOVA followed by Tukey's post hoc test. Asterisks (*) indicate significant differences among different groups (*p < 0.05, **p < 0.01, *p < 0.001).

To investigate whether elevated BCAAs contribute to exacerbated neuroinflammation, we first established an in vitro neuroinflammation model by exposing BV2 microglial cells to 1 µg mL^−1^ LPS. LPS stimulation markedly increased the release of pro‐inflammatory cytokines IL‐6 and IL‐1β in BV2 cells compared with the control group. Treatment with BCAAs alone did not significantly alter cytokine levels relative to controls (Figure 10A,B). Interestingly, co‐treatment with low (1 mM) and medium (2 mM) doses of exogenous BCAAs significantly attenuated LPS‐induced IL‐6 and IL‐1β secretion, whereas high‐dose (5 mM) BCAAs further potentiated the release of these pro‐inflammatory cytokines (Figure 10A,B). Western blot analysis revealed that LPS induced robust phosphorylation of NF‐κB, STAT3, and Akt, and high‐dose BCAAs in combination with LPS further potentiated this activation (Figure 10C–H). These results suggest that BCAAs may exert a dose‐dependent modulatory effect on microglial inflammation, with excessive BCAAs potentially aggravating inflammatory responses, possibly through Akt/STAT3/NF‐κB signaling, although causality requires further verification.

To further model neuronal injury‐associated inflammatory responses, SH‐SY5Y cells were exposed to 500 µM MPP^+^ to establish an in vitro PD‐like model. MPP^+^ significantly upregulated *Il‐6* and *Il‐1β* mRNA expression, indicating successful induction of a neuroinflammatory‐like response. Notably, supplementation with high‐dose BCAAs further increased the expression of these cytokines compared with MPP^+^ alone, suggesting that high‐dose BCAAs may exacerbate PD‐like neuronal injury‐associated inflammatory responses (Figure 10I,J). Mechanistically, high‐dose BCAAs appeared to amplify MPP^+^‐induced neuroinflammation, at least in part, via activation of the Akt/STAT3/NF‐κB signaling pathways (Figure 10K–P). Collectively, these findings indicate that BCAAs may exert bidirectional, dose‐dependent effects on neuroinflammation in vitro, with excessive BCAAs potentially exacerbating inflammatory responses in both microglial and neuronal cells. However, these observations are limited to in vitro models, and further studies are needed to confirm their relevance in vivo.

### 2.10 MPTP‐Induced Alterations in Gut Microbiota Impair the Intestinal Barrier and Promote Neuroinflammation

**FIGURE 11:**
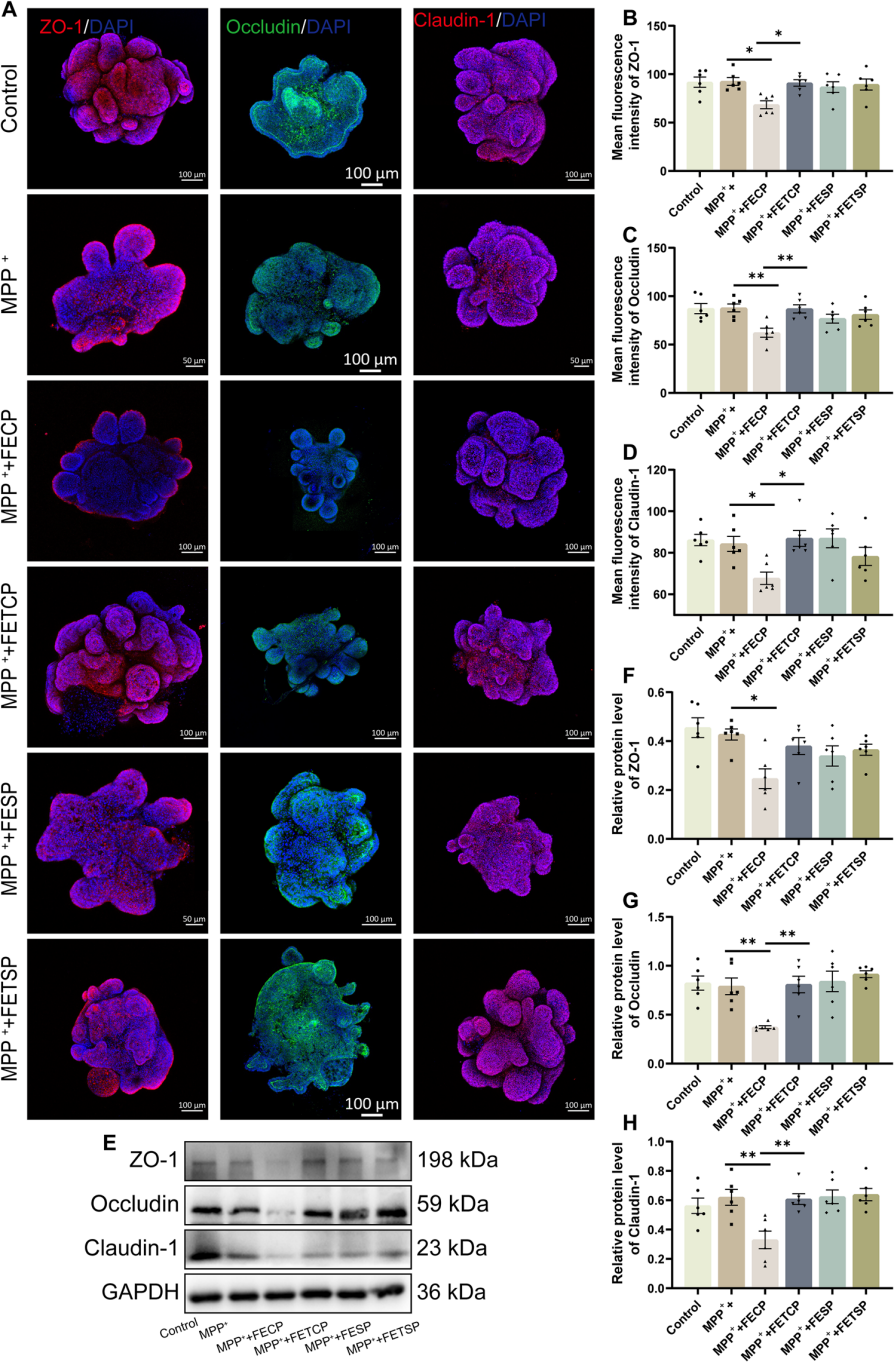
MPTP‐induced alterations in gut microbiota impair the intestinal barrier in mouse colonic organoids. (A) Representative IF images of ZO‐1 (red), Occludin (green) and Claudin‐1 (red) in the colon organoids. (B–D) Quantification of the mean fluorescence density of ZO‐1, Occludin and Claudin‐1 in the colon organoids (n = 6). (E) Representative western blotting of ZO‐1, Occludin, and Claudin‐1 protein in the colon organoids. (F–H) Quantification of ZO‐1, Occludin, and Claudin‐1 protein expression in the colon organoids (n = 6). FECP: fecal extract from casein‐fed PD mice; FETCP: fecal extract from TRF‐treated casein‐fed PD mice. FESP: fecal extract from soy protein‐fed PD mice; FETSP: fecal extract from TRF‐treated soy protein ‐fed PD mice. Error bars represent the mean ± SEM. Statistical significance for multiple group comparisons was determined using one‐way ANOVA followed by Tukey's post hoc test. Asterisks (*) indicate significant differences among different groups (*p < 0.05, **p < 0.01, *p < 0.001).

To elucidate the role of gut microbiota in MPTP‐induced intestinal barrier disruption and the protective effects of TRF, we co‐cultured fecal extracts from PD mice and TRF‐treated PD mice with mouse colonic organoids to mimic in vivo conditions and assessed their impact on the intestinal barrier. The results revealed that MPP^+^ treatment did no significant effect on the mean fluorescence intensity or protein expression of ZO‐1, Occludin, or Claudin‐1 in mouse colonic organoids. However, co‐treatment with MPP^+^ and fecal extracts from casein‐fed PD mice led to a significant reduction in the mean fluorescence intensity and protein expression of these tight junction proteins. In contrast, when MPP^+^ was combined with fecal extracts from TRF‐treated casein‐fed PD mice, no significant changes were observed in the mean fluorescence intensity or protein expression of ZO‐1, Occludin, or Claudin‐1. Notably, co‐treatment with MPP^+^ and fecal extracts from soy protein‐fed PD mice or TRF‐treated soy protein‐fed PD mice similarly showed no significant effects on the aforementioned tight junction proteins (Figure 11). These suggest that dietary protein sources (casein vs. soy) and microbial metabolites influence the impact of MPP^+^ on intestinal barrier integrity, with fecal extracts from casein‐fed PD mice exacerbating tight junction disruption caused by MPP^+^. The differential effects may be attributed to variations in gut microbial composition or metabolite profiles induced by distinct dietary proteins. In contrast, TRF preserves barrier integrity, potentially by modulating the gut microbiota.

To further investigate the impact of gut microbiota on inflammatory responses, fecal extracts from different groups were applied to inflammatory and neuronal cells. The results showed that fecal extracts from PD mice fed either casein or soy protein further exacerbated LPS‐induced cytokine release (IL‐1β and IL‐6), indicating a pronounced pro‐inflammatory effect of PD‐associated gut microbiota. In contrast, fecal extracts from TRF‐treated PD mice, regardless of whether they were fed casein or soy protein, significantly reduced LPS‐induced IL‐1β and IL‐6 levels. These findings suggest that TRF may attenuate inflammatory responses by modulating the gut microbiota or their metabolites, and that the anti‐inflammatory effect is not influenced by dietary protein source (Figure S4). These findings highlight the critical role of gut microbiota and their metabolites in modulating the gut‐brain axis.

### 2.11 Allobaculum Mucilyticum Induces Intestinal Barrier Impairment in Mouse Colonic Organoids

**FIGURE 12:**
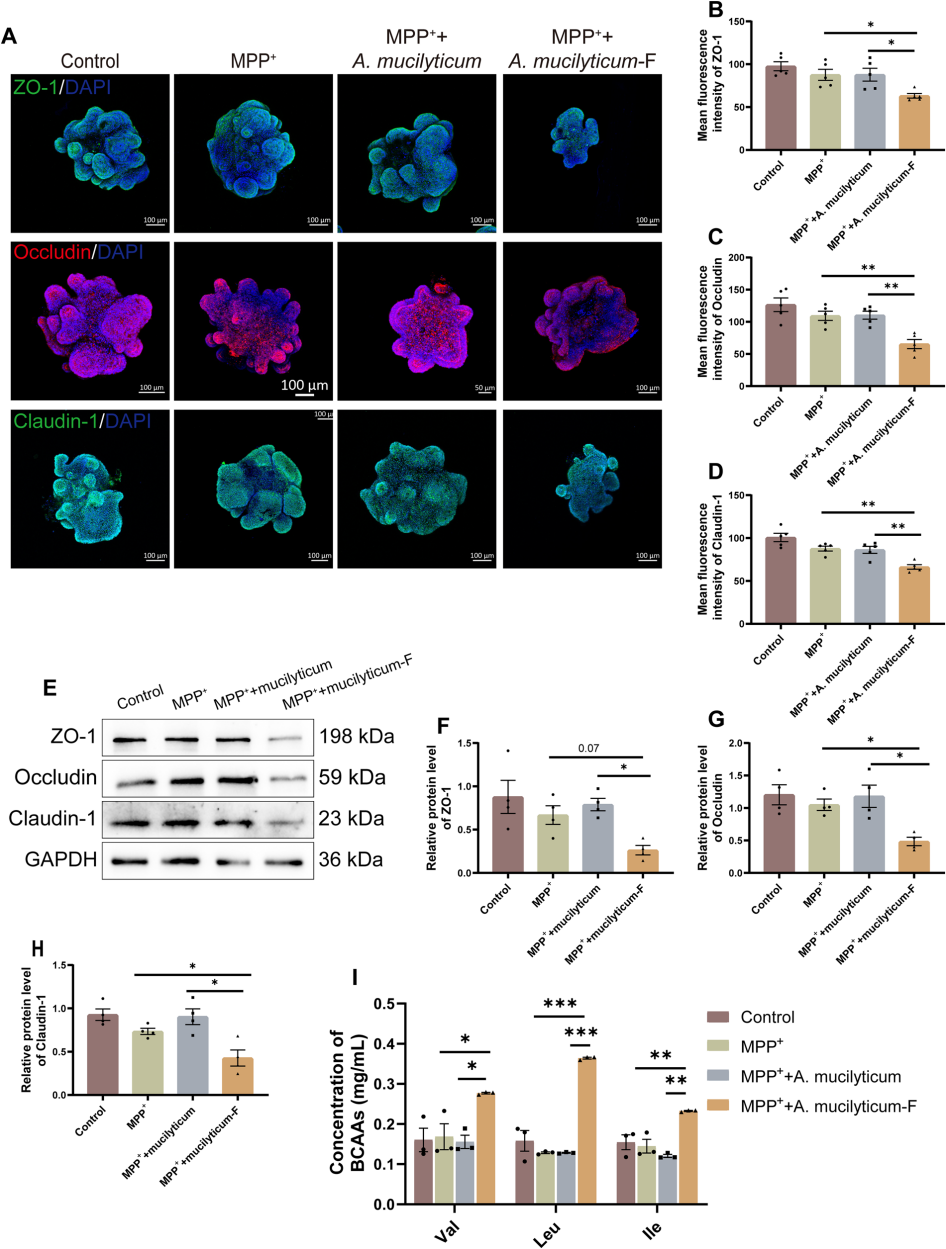
*Allobaculum mucilyticum* induces intestinal barrier disruption in mouse colonic organoids. (A) Representative IF images of ZO‐1 (green), Occludin (red), and Claudin‐1 (green) in the colon organoids. Scale bar: 100 µm. (B–D) Quantification of the mean fluorescence density of ZO‐1, Occludin and Claudin‐1 in the colon organoids (n = 5). (E) Representative western blotting of ZO‐1, Occludin and Claudin‐1 protein in the colon organoids. (F–H) Quantification of ZO‐1, Occludin, and Claudin‐1 protein expression in the colon organoids (n = 4). (I) Concentration of BCAAs in culture medium after colon organoids were cultured with the indicated treatment (n = 3). *A. mucilyticum*‐F: fermentation broth of *A. mucilyticum*. Error bars represent the mean ± SEM. Statistical significance for multiple group comparisons was determined using one‐way ANOVA followed by Tukey's post hoc test. Asterisks (*) indicate significant differences among different groups (*p < 0.05, **p < 0.01, *p < 0.001).

MPTP treatment markedly increased in the abundance of *Allobaculum* in casein‐fed PD mice, an effect reversed by TRF. *Allobaculum mucilyticum* (*A. mucilyticum*), isolated from the feces of individuals with inflammatory bowel disease, has been implicated in intestinal mucus layer degradation [21, 22]. To determine whether *A. Mucilyticum* may contributes to colonic barrier dysfunction, we co‐treatment with MPP^+^ and with either *A. mucilyticum* or its fermentation broth (*A. mucilyticum*‐F) mouse colonic organoids. Notably, *A. mucilyticum* fermentation broth (*A. mucilyticum*‐F) significantly reduced the mean fluorescence intensity and protein expression of ZO‐1, occludin, and claudin‐1 in colonic organoids, while increasing the concentration of BCAAs in the culture medium (Figure 12A‐H). In contrast, *A. mucilyticum* itself had no significant effect (Figure 12I). These findings indicate that *A. mucilyticum* does not directly compromise the intestinal barrier, but may induce barrier impairment through its metabolic products.

## 3 Discussion

Dietary factors and gut health have attracted growing attention in PD research, yet most studies have focused on either specific nutrients or feeding patterns in isolation. The combined impact of nutrient composition and temporal feeding remains largely underexplored. In this study, we systematically investigated how different protein sources (casein vs. soy protein) and TRF interact to influence PD‐related symptoms in the MPTP‐induced mouse model, aiming to identify dietary strategies that mitigate disease progression.

Soy protein, a complete plant‐based protein rich in essential amino acids, is widely consumed by vegetarians. Although soy isoflavones have been reported to ameliorate motor deficits in PD models and are associated with reduced mortality in observational studies [23, 24], the specific effects of soy protein itself remain poorly understood. Here, we demonstrate that soy protein partially alleviates MPTP‐induced motor deficits, gut inflammation, and barrier disruption, despite the persistence of dopaminergic neuronal loss and neuroinflammation. These effects may be attributed to the intrinsic properties of soy protein. Soy protein contains higher levels of bioactive peptides and isoflavones compared with casein, which have been reported to modulate gut microbiota, enhance tight junction protein expression, and suppress proinflammatory cytokine production [25, 26]. Additionally, soy protein is richer in arginine and certain essential amino acids, and exhibits moderate digestibility, whereas casein is slow‐digesting and has a higher content of BCAAs [27]. Dietary soy protein concentrate improves intestinal barrier function and attenuates DSS‐induced colitis by suppressing NLRP3 inflammasome activation and caspase‐1 activity [28], and soy protein isolates have been shown to differentially regulate intestinal mucin production, immunoglobulin secretion, and immune signaling in early‐development mice compared with casein [29]. Together, our findings suggest that soy protein may exert beneficial effects on gut health, which may contribute to the partial mitigation of PD‐related symptoms observed in our study.

In contrast, casein‐fed PD mice exhibited more severe gut barrier damage, potentially due to the “slow‐digesting” nature of casein and higher BCAAs content, which delays gastric emptying and reduces intestinal motility [30]. This finding is consistent with epidemiological evidence linking high dairy intake to an increased risk of PD [31]. Although intestinal barrier dysfunction has been reported in subacute and chronic MPTP models [32, 33], its presence in acute models has been less well characterized. Our findings highlight, for the first time, that even in acute MPTP models, dietary protein source significantly impacts gut pathology. Collectively, these results suggest that soy protein may offer a protective advantage over animal‐derived casein in PD.

TRF, an eating pattern that aligns food intake with circadian rhythms without restricting calories, has shown promise in metabolic and neurodegenerative disease models [34, 35]. However, the potential influence of dietary composition on the efficacy of TRF has been largely overlooked. For example, shift workers exhibit a higher risk of metabolic syndrome (RR = 1.57, 95% CI = 1.24–1.98) [36, 37, 38], potentially due to a higher intake of unhealthy foods and shorter fasting durations in shift workers compared to non‐shift workers (11.8 h ± 2.0 h vs. 13.3 h ± 1.9 h) [39]. These findings underscore that both what and when we eat are crucial for health maintenance. In this study, we observed that TRF significantly attenuated neuroinflammation regardless of protein source, but the combination of TRF and soy protein yielded greater neuroprotection and promoted dopaminergic neuron recovery. Although TRF exerted comparable anti‐inflammatory effects in casein‐ or soy protein‐fed PD mice, its superior protection of dopaminergic neurons in soy protein‐fed PD mice suggests that mechanisms beyond inflammation are involved. Soy‐derived bioactive peptides and isoflavones have been shown to enhance mitochondrial respiration, upregulate antioxidant enzymes (e.g., superoxide dismutase, glutathione peroxidase), and suppress reactive oxygen species generation [40, 41], thereby improving redox balance and cellular energy metabolism. Moreover, isoflavones such as genistein can activate autophagic flux via AMPK‐mTOR signaling, facilitating the clearance of misfolded or aggregated proteins [34, 42]. These mitochondrial‐ and autophagy‐related effects may synergize with the circadian alignment of TRF to reinforce neuronal resilience and delay neurodegeneration. Together, these findings imply that the neuroprotective efficacy of TRF can be modulated by dietary composition, and that combining TRF with a favorable protein source such as soy may maximize its therapeutic potential in PD.

Considering the rapid (within 24 h) impact of diet on the gut microbiota and the crucial role of gut dysbiosis in PD [43, 44], an important question arises: Is the protective effect of TRF in PD mediated through alterations in the gut microbiota? Our analysis of gut microbial composition and metabolites after four weeks of intervention provides insight into this possibility. Notably, microbiome analysis reveals that dietary protein sources distinctly shape gut microbiota composition in PD mice.

*Allobaculum*, a gram‐positive anaerobe from the *Erysipelotrichaceae* family, is notably influenced by dietary protein source. In our study, *Allobaculum* was significantly enriched in casein‐fed PD mice but markedly reduced following TRF intervention. A related species, *A. mucilyticum*, a mucin‐degrading bacterium isolated from IBD patients, has been shown to exacerbate colitis [45, 46]. In our study, fermentation products of *A. mucilyticum* disrupted the integrity of colonic organoid barriers, supporting the notion that *Allobaculum* may aggravate PD‐associated gut barrier damage through its metabolic products. These findings provide mechanistic insight into how TRF may restore gut function in PD mice by modulating microbial composition.

The composition of the gut microbiota critically shapes its functional output. PICRUSt functional predictions and metabolomics analyses revealed that casein‐fed PD mice exhibited abnormally elevated BCAAs in both serum and feces, a dysregulation effectively reversed by TRF. This aligns with previous findings of elevated BCAAs in PD patients and MPTP‐induced PD models [47, 48]. Our recent study revealed that excessive BCAAs impair microglial clearance of α‐synuclein in gut‐originated PD models by inducing lysosomal dysfunction [49], implicating excessive BCAAs as a contributor to PD pathogenesis. Consistent with this, our cell‐based experiments confirmed that exogenous BCAAs, via activation of the NF‐κB/Akt/STAT3 signaling pathway, not only exacerbated LPS‐induced neuroinflammation in BV2 cells, but also enhanced MPP^+^‐induced PD‐like neuronal injury‐associated inflammatory responses in SH‐SY5Y cells. Building upon these insights, our current study shows that TRF may promote BCAAs catabolism, thereby mitigating neuroinflammation in the context of a casein‐rich diet. Mechanistically, TRF modulated microbial metabolism by reducing the abundance of *Allobaculum* and enhancing BCAAs degradation, which collectively alleviated gut barrier disruption and neuroinflammation. Although TRF did not restore dopaminergic neuron loss, it significantly improved partially motor function in casein‐fed PD mice, consistent with previous findings showing that modulation of DA metabolism [50], or inhibition of neuroinflammation [51] can enhance motor performance independently of dopaminergic neuron survival.

Unlike dietary casein, soy protein promotes a gut microbial environment enriched with *Akkermansia muciniphila*, a mucin‐degrading bacterium recognized as a next‐generation probiotic. The combination of soy protein and TRF can alleviate MPTP‐induced reduction of *Akkermansia*, suggesting a gut‐brain axis mechanism underlying the observed neuroprotection. Previous studies have demonstrated tha*t Akkermansia muciniphila* ameliorates MPTP‐induced motor dysfunction and dopaminergic neurons loss by increasing isovaleric acid levels [52]. Interestingly, although soy protein‐fed PD mice did not exhibit BCAAs dysregulation, they showed a relative reduction in fecal SCFAs, which was reversed by TRF. This contrasts with the casein‐fed condition, where SCFAs remained persistently low, highlighting a diet‐dependent SCFA profile in PD. Given that *Akkermansia muciniphila* produces SCFAs such as valeric and propionic acid‐metabolites known to promote beneficial microbes, reinforce gut barrier function, and modulate inflammatory cytokines including IL‐6 and TNF‐α [53]‐ the restoration of SCFAs by TRF likely contributes to its neuroprotective effect. Collectively, these findings suggest that, under a soy protein diet, TRF enhances *Akkermansia muciniphila* abundance and SCFA production, thereby alleviating dopaminergic neuron loss and improving motor performance in PD mice through a microbiota‐mediated mechanism.

### 3.1 Limitations and Future Directions

We acknowledge several limitations of the present study. First, the use of an acute MPTP‐induced mouse model may not fully recapitulate the progressive and multifactorial pathology of human PD. Future studies could employ progressive PD models, such as α‐synuclein preformed fibril inoculation models, which better mimic the gradual neurodegeneration and gut‐brain axis alterations observed in patients. Second, only one sex of animals was used in this study, which may limit the generalizability of the findings. Future experiments including both male and female mice would help to clarify potential sex‐dependent effects of TRF. Third, although this study revealed correlations between dietary protein source, gut microbiota, and host metabolism, direct causal relationships were not established. Future research incorporating microbiota transplantation, BCAA supplementation, or metabolite intervention approaches will be essential to determine the mechanistic basis of these associations. In addition, clinical investigations will be valuable to validate the translational potential of these findings in human PD populations.

## 4 Conclusion

In summary, our findings suggest that both dietary composition and meal timing may play important roles in the progression of PD. Under a casein‐based diet, TRF mitigates gut barrier integrity and neuroinflammation by reducing *Allobaculum* abundance and promoting BCAAs catabolism, which may together contribute to alleviated motor dysfunction. In contrast, under a soy protein‐based diet, TRF is associated with enhanced dopaminergic neuron survival and attenuated neuroinflammation, possibly through by enriching *Akkermansia muciniphila* and promoting the production of its key metabolites, SCFAs. Importantly, compared to the animal‐derived casein diet, the plant‐based soy protein diet more effectively enhances the neuroprotective effects of TRF in the MPTP‐induced PD model, as reflected by improved dopaminergic neuron survival. These results suggest that combining TRF with plant‐based protein diets may represent a promising dietary strategy for PD management. Future studies, including in‐depth investigations into gut‐brain axis mechanisms and clinical trials, are needed to validate the therapeutic potential of TRF combine with plant‐based protein diets for PD.

## 5 Experimental Section

### 5.1 Animals

Six‐week‐old male C57BL/6J mice (weighing 18 ± 2 g) were purchased from the Gem Pharmatech (Nanjing, China). The mice were housed in a pathogen‐free environment at 24 ± 2.0°C with controlled humidity (55 ± 10%) and 12 h light/dark cycles. Lights‐on time was defined as zeitgeber time 0 (ZT 0) and lights‐off time as ZT 12 (lights on between 7:00 and 19:00). Before the experiments, the animals were adapted for a week during which they were free to obtain the standard diet and sterile water. All experimental procedures were approved by the Animal Ethics Committee of Jiangnan University (JN.No20211215c1600717[579]).

### 5.2 Diets and Group Design

The casein‐based diet was formulated according to the AIN‐93G standard, while the soy protein‐based diet was prepared by substituting casein in the AIN‐93G formula with soy protein (Jiangsu Xietong Organism, China). The ingredients and nutrient composition of the experimental diets are listed in Table 1. After a one‐week acclimatization period, mice were randomly assigned to two dietary groups (casein or soy protein). Each dietary group was further divided into four subgroups (eight groups in total): (1) Control group: Mice had ad libitum access to food. (2) TRF group: Mice had food access from ZT 13 (1 h after lights off) to ZT 21 (3 h before lights on) during their active phase. (3) MPTP group: Mice had food access restricted to ZT 13‐ZT 21 during their active phase. (4) TRF+MPTP group: Mice had food access during the active phase and received intraperitoneal injections of MPTP. A schematic of the experimental design is shown in Supplementary Figures 1A and 2A. All analyses were conducted on a single experimental dataset generated from this design. Different predefined group comparisons were analyzed as separate analytical endpoints, including effects related to dietary protein source and the modulatory effects of TRF under different dietary conditions. Body weight and food intake were recorded throughout the feeding period.

**TABLE 1:** Composition of AIN‐93G‐based casein and soy protein diets.

Nutrient compositions	Casein		Soy	
	gm%	kcal%	gm%	kcal%
Protein	20.30%	20.30%	20.30%	20.30%
Carbohydrate	63.95%	63.95%	63.95%	63.95%
Fat	7.00%	15.75%	7.00%	15.75%
Total		100.00%		100.00%
kcal gm^−1^	4.00		4.00	
	Casein		Soy	
**Ingredient**	gm	kcal	gm	kcal
Casein	200	800	0	0
Soy protein	0	0	200	800
L‐Cystine	3	12	3	12
Corn Starch	397.5	1590	397.5	1590
Maltodextrin	132	528	132	528
Sucrose	100	400	100	400
Cellulose	50	0	50	0
Soybean Oil	70	630	70	630
Vitamin Mix V10037	10	40	10	40
Mineral Mix S10022G	35	0	35	0
Choline Bitartrate	2.5	0	2.5	0
Total	1000	4000	1000	4000
**BCAAs content** **[g kg^−1^]**	Casein		Soy	
Valine	22.96		14.28	
Leucine	34.32		24.92	
Isoleucine	18.64		14.64	
Total	75.92		53.84	

### 5.3 MPTP Treatment

MPTP is widely recognized for inducing destruction of the nigrostriatal dopaminergic pathway and replicating key symptoms of PD, making it the most commonly used model for PD research. To establish an acute PD mouse model, MPTP (MO896, Sigma‐Aldrich, USA; 15 mg kg^−1^) was dissolved in saline and administered intraperitoneally at a volume of 10 mL kg^−1^ body weight, four times at 2 h intervals on day 21 of the experiment. Behavioral assessments were conducted, and tissues were collected on the seventh day after MPTP injection, by which time the reduction in striatal dopamine levels and the loss of dopaminergic neurons had reached a stable level.

### 5.4 Behavioral Tests

Behavioral assessments were conducted on day 28 using a double‐blind design.

Pole test: A 50 cm‐long, 1 cm‐diameter pole wrapped in non‐adhesive gauze for grip, with a 2 cm spherical top, was placed in the home cage. Mice were positioned head‐up at the top, and descent time was recorded. Each mouse underwent three trials with 15 min intervals, and the average descent time was calculated, which serves as an indicator of motor coordination and bradykinesia.

Traction test: Mice were placed on a 5 mm‐diameter horizontal rope by their forepaws, and their ability to grasp the rope with all limbs was observed for 10 s. Scoring ranged from 1 to 4, with lower scores indicating greater impairment: all four limbs grasping (4), one hind limb grasping (3), both forelimbs grasping (2), and one forelimb grasping (1). Each mouse underwent three trials with 15 min intervals, and the average score was calculated, which serves as an indicator of basic motor coordination deficits.

### 5.5 Sample Collection and Tissue Preparation

On the morning of day 28, mice were individually placed in autoclaved cages for fecal collection. Once defecated, feces were immediately transferred to sterile EP tubes, placed on ice, and stored at ‐80°C for gut microbiota profiling, free amino acid analysis, and SCFAs assays. After deep anesthesia with isoflurane (3%), blood was collected via orbital puncture, centrifuged at 3,000 rpm for 15 min, and the serum was stored for free amino acid analysis. Fresh striatal and colonic tissues were rapidly dissected and stored at ‐80°C for subsequent analysis. For frozen brain sections, mice were transcardially perfused with cold saline followed by 4% paraformaldehyde (PFA) in 0.01 M phosphate‐buffered saline (PBS). Brains were post‐fixed in 4% PFA at 4°C overnight, cryoprotected sequentially in 20% and 30% sucrose solutions (24 h each), and embedded in optimal cutting temperature (O.C.T.) compound. Coronal brain sections (10 µm thick) were prepared using a cryostat microtome (CM1950, Leica, Germany), and regions containing the striatum (Bregma 0.14 to 1.34 mm) and SNpc (Bregma ‐2.92 to ‐3.52 mm) were collected. For paraffin‐embedded sections, the distal colon was fixed, dehydrated through graded alcohols, embedded in paraffin, and sectioned at 4 µm thickness using a paraffin microtome (1150H, Leica, Germany).

### 5.6 Measurement of Neurotransmitters

High‐performance liquid chromatography (HPLC) with a fluorescence detector (Waters 2475, Milford, MA, USA) was employed to measure the levels of striatal DA, and its metabolites, including DOPAC, and HVA. Briefly, the striatum was homogenized in 0.1 M perchloric acid (10 µL mg^−1^ of tissue) by sonication, and the homogenate was centrifuged at 13000 rpm, 4°C for 10 min. The supernatants were then collected and filtered through a 0.22‐µm filter. Calibration curves were constructed using standards of DA (Sigma‐Aldrich, #PHR1090), DOPAC (Sigma‐Aldrich, #11569), and HVA (Sigma‐Aldrich, #69673), dissolved in 0.1 M perchloric acid and diluted to concentrations of 0.01, 0.02, 0.04, 0.08, 0.1, 0.2, 0.4, 0.8, 1, 1.5, 2, 4, 8, 10, and 20 µg mL^−1^. Both samples and standards were analyzed using a Waters 2695 HPLC system with an Atlantis T3 column (150 mm × 4.6 mm, 5 µm, Waters). The mobile phases consisted of water, acetonitrile, and 0.01 M PBS (pH 4.0). The injection volume was 25 µL, and the total run time for each analysis was 23 min. The concentrations of the target neurotransmitters in the striatum were determined using an external standard method.

### 5.7 Western Blotting Analysis

The striatum and distal colon tissues were homogenized to extract total protein using a mixture of radio immunoprecipitation assay lysis buffer (RIPA, P0013C, Beyotime, China), 1% phenylmethanesulfonyl fuoride (PMSF, ST506, Beyotime, China) and 2% phosphatase inhibitor (P1081, Beyotime, China). The homogenate underwent centrifugation at 13,000 rpm at 4°C for 15 min, after which the supernatant was collected. Total protein concentration was determined using a BCA Protein Quantification Kit (E112‐02, vazyme, China). Following boiling denaturation, 25 µg of total protein was separated via 10% sodium dodecyl sulfate‐polyacrylamide gel electrophoresis and transferred onto a polyvinylidene fluoride membrane (ISEQ00010, Millipore, USA). The membrane was then blocked with 5% (w/v) skim milk (36120ES76, Yeasen Biotech, China) in Tris‐buffered saline containing 0.1% Tween‐20 (P1379, Sigma‐Aldrich, USA) (TBST) for 2 h at room temperature. Each membranes were separately incubated overnight at 4°C with specific antibodies: rabbit anti‐TH (1:1000, MAB152, Millipore, USA), rabbit anti‐MAO‐B (1:1000, 12602‐1‐AP, Proteintech, China), rabbit anti‐ZO‐1 (1:1000, 21773‐1‐AP, Proteintech, China), rabbit anti‐Occludin (1:1000, 27260‐1‐AP, Proteintech, China), rabbit anti‐Claudin‐1 (1:200, TA0127, Abmart, China), rabbit anti‐p‐NF‐κB (Ser536, 1:1000, 3033, Cell Signaling Technology, USA), rabbit anti‐NF‐κB (1:1000, 8242, Cell Signaling Technology, USA), rabbit anti‐p‐STAT3 (Tyr705, 1:1000, 9145, Cell Signaling Technology, USA), rabbit anti‐STAT3 (1:1000, 4904, Cell Signaling Technology, USA), rabbit anti‐p‐Akt (Ser473, 1:1000, 4060, Cell Signaling Technology, USA), rabbit anti‐Akt (1:1000, 4691, Cell Signaling Technology, USA) and rabbit anti‐GAPDH (1:8000, 10494‐1‐AP, Proteintech, China). After washing 3 times with TBST for 10 min each at room temperature, goat anti‐rabbit IgG (1:8000, BA1054, Boster, China) conjugated to horseradish peroxidase were used as secondary antibody and incubated for 2 h at room temperature. Subsequently, after another three washes with TBST for 10 min each at room temperature, the protein bands were visualized using an enhanced chemiluminescence reagent (36208ES60, Yeasen, China) and imaged with a Gel Image System (Tanon‐5200Multi, China). Target signals were quantified by using Image J software (NIH, Bethesda, USA).

### 5.8 Immunofluorescence

The sections were immersed in 0.01 M sodium citrate buffer (pH 6.0) for antigen retrieval at 95°C and then washed thrice in PBS. Furthermore, brain sections were incubated in PBS containing 0.3% v/v Triton X‐100 and 10% v/v goat serum for 30 min at 37°C. The primary antibodies: mouse anti‐TH (1:1000, GB12181, Servicebio, China), rabbit anti‐GFAP (1:500, GB11096, Servicebio, China) and rabbit anti‐Iba‐1, (1:500, GB113502, Servicebio, China), rabbit anti‐MAO‐B (1:200, 12602‐1‐AP, Proteintech, China), rabbit anti‐ZO‐1, (1:1000, 21773‐1‐AP, Proteintech, China), rabbit anti‐Occludin (1:1000, 27260‐1‐AP, Proteintech, China), rabbit anti‐Claudin‐1 (1:200, 28674‐1‐AP, Proteintech, China) were incubated overnight at 4°C. Secondary antibodies coupled to FITC‐conjugated goat anti‐mouse IgG (1:1000, A0568, Beyotime, China) or Cy3‐conjugated goat anti‐rabbit IgG (1:1000, A0516, Beyotime, China). After a final wash with PBS, the sections were mounted onto glass slides, and Antifade Mounting Medium with DAPI (P0131, Beyotime, China) was applied for visualization of the nuclei. Fluorescence imaging was d performed using a laser confocal microscope (Carl Zeiss LSM880, Zeiss, Germany). Quantitative analysis of TH‐positive neurons, GFAP‐positive astrocytes, and Iba‐1‐positive microglia in the SNpc was conducted using ImageJ software (version 1.53k, NIH, Bethesda, MD, USA). For each mouse, we analyzed five coronal midbrain sections: the section containing the maximal SNpc area, together with two anterior sections and two posterior sections, each spaced 100 µm apart. The number of TH‐, GFAP‐, and Iba‐1‐positive cells in the SNpc was calculated as the number of positive cells per square millimeter (cells/mm^2^).

### 5.9 Enzyme‐Linked Immunosorbent (ELISA) Assay

The protein level of IL‐6, and IL‐1β in the striatum ang colon were detected by a commercial mouse IL‐6 ELISA kit (EK0411, Boster, China), and IL‐1β ELISA kit (DY401, R&D, USA). The ELISA procedure was carried out in accordance with the manufacturer's instructions. Absorbance was determined at 450 nm using Synergy MX (BioTek Instruments, Inc., USA).

### 5.10 Quantitative Real‐Time PCR (qRT‐PCR) Analysis

To extract total RNA from the striatum, RNA isolater Total RNA Extraction Reagent (R401‐01, vazyme, China) was utilized. Subsequently, 1000 ng of total RNA underwent reverse transcribed into cDNA using PrimeScript RT Kit (RR036A, TaKaRa, Japan). PCR was conducted using SYBR Pre‐mix Ex TaqTM II (RR820A, TaKaRa, Japan) on the Light‐Cycler 480 II (Roche, Switzerland). The relative expression of target genes was calculated by the 2^−ΔΔCt^ method and normalized to GAPDH. The primer sequences were listed in Table 2.

**TABLE 2:** Sequences of the gene‐specific primers.

	Forward primer	Reverse primer
*Il‐4*	GGTCTCAACCCCCAGCTAGT	GCCGATGATCTCTCTCAAGTGAT
*Il‐10*	GCTCTTACTGACTGGCATGAG	CGCAGCTCTAGGAGCATGTG
*Bcat1*	GAAGTGGCGGAGACTTTTAGG	TGGTCAGTAAACGTAGCTCCA
*Bcat2*	AAAGCATACAAAGGTGGAGACC	CGTAGAGGCTCGTTCCGTTG
*Bckdk*	ACATCAGCCACCGATACACAC	GAGGCGAACTGAGGGCTTC
*Il‐6*	CTGCAAGAGACTTCCATCCAG	AGTGGTATAGACAGGTCTGTTGG
*Il‐1β*	GAAATGCCACCTTTTGACAGTG	TGGATGCTCTCATCAGGACAG
*Gapdh*	TGGAGAAACCTGCCAAGTATGA	TGGAAGAATGGGAGTTGCTGT

### 5.11 Gut Microbiota Profiling

Bacterial genomic DNA from fecal samples was immediately extracted using the MagBeads FastDNA Kit for Soil (116564384, MP Biomedicals, CA, USA) according to the manufacturer's protocol, and stored at ‐20°C prior to further analysis. To ensure successful DNA isolation, the quantity and quality of extracted DNAs were measured using a NanoDrop NC2000 spectrophotometer (Thermo Fisher Scientific, Waltham, MA, USA) and agarose gel electrophoresis, respectively. The V3‐V4 region of the bacterial 16S rRNA was amplified by PCR using primers 338F (5’‐ACTCCTACGGGAGGCAGCA‐3’) and 806R (5’‐GGACTACHVGGGTWTCTAAT‐3’). Sample‐specific 7‐bp barcodes were incorporated into the primers for multiplex sequencing. The PCR components contained 5 µl of buffer (5×), 0.25 µL of Fast pfu DNA Polymerase (5U µL^−1^), 2 µL (2.5 mM) of dNTPs, 1 µL (10 µM) of each Forward and Reverse primer, 1 µL of DNA Template, and 14.75 µL of ddH2O. Thermal cycling consisted of initial denaturation at 98°C for 5 min, followed by 26 cycles consisting of denaturation at 98°C for 30 s, annealing at 52°C for 30 s, and extension at 72°C for 45 s. After cycling, a final extension at 72°C for 5 min was performed, and the reaction was then kept at 12°C. PCR amplicons were purified using Vazyme VAHTSTM DNA Clean Beads (Vazyme, Nanjing, China) and quantified using the Quant‐iT PicoGreen dsDNA Assay Kit (Invitrogen, Carlsbad, CA, USA). After the individual quantification step, amplicons were pooled in equal amounts, and pair‐end 2 × 250 bp sequencing was performed using the Illlumina NovaSeq platform with NovaSeq 6000 SP Reagent Kit (500 cycles) at Shanghai Personal Biotechnology Co., Ltd (Shanghai, China).

Sequence data analyses were mainly performed using QIIME2 [38] and R packages (v3.6.0). The α‐diversity and β‐diversity analyses were performed to investigate the structural variation of microbial communities across samples using UniFrac distance metrics and visualized via PCoA. Differences in the UniFrac distances among groups were determined by ANOSIM. PICRUSt based on OTUs was employed to predict the abundances of functional categories using KEGG orthologs.

### 5.12 Metabolomics Profiling of Serum Samples

Serum samples were separated by centrifugation and stored at −80°C until analysis. For metabolite extraction, samples were thawed on ice, vortexed, and precipitated with methanol. After centrifugation, the supernatant was collected, dried, reconstituted in 80% methanol containing an internal standard, filtered, and subjected to LC‐MS analysis.

Untargeted metabolomics was performed on a Vanquish UHPLC system coupled with a Q Exactive Orbitrap mass spectrometer (Thermo Fisher Scientific, USA). Chromatographic separation was achieved using an ACQUITY UPLC HSS T3 column (2.1 × 100 mm, 1.8 µm) under both positive and negative electrospray ionization modes. Gradient elution was conducted with water and acetonitrile containing 0.1% formic acid or 5 mM ammonium format as mobile phases [54, 55].

Raw data were converted to the mzXML format using MSConvert and processed in R with the XCMS package for peak detection, retention time correction, and alignment. Features with RSD > 30% in quality control samples were excluded. Metabolite identification was performed by matching accurate mass and MS/MS spectra against multiple databases, including HMDB, MassBank, KEGG, LipidMaps, mzCloud, and an in‐house library.

Multivariate analyses (PCA, PLS‐DA, OPLS‐DA) were performed using the ropls package. Differential metabolites were identified based on VIP > 1 and *p* < 0.05, and pathway enrichment analysis was conducted using the KEGG database to identify significantly affected metabolic pathways.

### 5.13 Detection of Free Amino Acids in Serum and Feces

Determination of free amino acids in serum and fecal samples by O‐phthalaldehyde precolumn derivatization and reversed‐phase HPLC. The serum samples were supplemented with an equal volume of 10% trichloroacetic acid (T433652, Aladdin, China) to attain a final concentration of 5%. After standing at 4°C for 30 min, the supernatant was harvested by centrifugation at 10 000 rpm at 4°C for 10 min. The feces samples were weighed, suspended in cold 5% trichloroacetic acid solution, and homogenized. Standing in a 4°C refrigerator for 30 min, and the supernatant was harvested by centrifugation at 15 000 rpm at 4°C for 30 min. The chromatography separation condition in HPLC‐MS/MS was achieved on an Agilent Hypersil ODS column (4.0 mm × 250 mm, 5 µm) at 40°C. The mobile phase A was 27.6 mM sodium acetate‐triethylamine‐tetrahydrofuran (500:0.11:2.5, vol/vol), and mobile phase B was 80.9 mM sodium acetate‐methanol‐acetonitrile (1:2:2, vol/vol), at a flow rate of 1 mL min^−1^. The gradient elution was set as mentioned: 0 min, 8% B; 17 min, 50% B; 20.1 min, 100% B; 24 min, 0% B. The content of the target free amino acids in serum and feces was determined using an external standard method.

### 5.14 Fecal SCFAs Profiling

Fecal SCFAs concentrations were determined using gas chromatography‐mass spectrometry (GC‐MS). Initially, 50 mg fecal pellets were mixed with 100 µL of ultrapure water and homogenized. f Subsequently, the mixture was centrifuged at 4°C at 14000 rpm for 15 min. The resulting supernatant was taken and acidified by adding 50 µL of 50% (v/v) sulfuric acid per 100 µL of supernatant. After vortexing and standing, the organic acids were extracted by adding 225 µL of n‐hexane, the supernatant was collected and filtered through a 0.22 µm organic filter. A mixture of acetic acid (A116165, Aladdin, China), propionic acid (P110443, Aladdin, China), isobutyric acid (I103521, Aladdin, China), butyric acid (B110439, Aladdin, China), isovaleric acid (I108280, Aladdin, China), and valeric acid (V108269, Aladdin, China) standards were dissolved in n‐hexane and diluted to a series of concentrations of 0.5, 1, 2, 5, 10, 20, 50, and 100 µg mL^−1^ to construct the calibration curves. Both samples and standards were analyzed using a Trace 1300/Exactive GC apparatus (Thermo Fisher Scientific, Waltham, MA, USA) equipped with flame electron impact ionization and an Rtx‐WAX column (30 m × 0.25 mm × 0.25 µm, Bellefonte, PA, USA). The initial temperature of the chromatographic column was held at 100°C for 1 min, and then raised to 140°C with ramping of 8°C min^−1^. Subsequently, the temperature was further increased to 200°C at a rate of 60°C min^−1^ and be sustained for 3 min. The injection volume was 1 µL and the total run time for a single analysis was 9 min. Hydrogen was used as the carrier gas at a flow rate of 47 mL min^−1^. The content of the target acids in feces was determined using an external standard method.

### 5.15 Cells Culture and Treatment

BV2 microglial cells (RRID:CVCL_0182; SCSP‐5053, Chinese Academy of Sciences), SH‐SY5Y neuroblastoma cells (RRID:CVCL_0019; SCSP‐5014, Chinese Academy of Sciences), and MA cells, were immortalized from mouse primary astrocytes (cat. no. 1800–57) provided by ScienCell Research Laboratories, Inc., were used in this study. Both cell lines were cultured in Dulbecco's Modified Eagle Medium (DMEM; PM150210, Pricella, China) supplemented with 10% fetal bovine serum (FBS; PYG0109, Boster, China) and 100 U mL^−1^ penicillin‐streptomycin (15140122, Gibco, USA), and maintained at 37°C in a humidified atmosphere containing 5% CO_2_.

For BV2 cells, neuroinflammation was induced by lipopolysaccharide (LPS; 1 µg mL^−1^, L2630, Sigma‐Aldrich, USA) for 24 h in the presence or absence of BCAAs (leucine:valine:isoleucine = 2:1:1; L8912, V1255, I7403; Sigma‐Aldrich, USA) at low (1 mM), medium (2 mM), or high (5 mM) concentrations.

SH‐SY5Y cells at passages 5–15 were differentiated with 10 µM all‐trans retinoic acid (RA; R2625, Sigma‐Aldrich, USA) in DMEM containing 2% FBS for 5 days to induce a neuron‐like phenotype before treatment. Differentiated cells were then exposed to 500 µM MPP^+^ (HY‐W008719, MedChemExpress, China) with or without 5 mM BCAAs for 24 h to evaluate the impact of elevated BCAAs on neuronal injury‐associated inflammatory responses.

### 5.16 Extraction of Feces and Cell Culture

A total of 100 mg fecal pellets were suspended in 1000 µL sterile PBS, smashed, and vortexed for 30 s to obtain a homogeneous suspension. The mixture was then centrifuged at 8000 rpm for 10 min, and the supernatant was collected and filtered through a 0.22 µm sterile syringe filter to remove bacteria and debris. BV2, MA, and SH‐SY5Y cells were treated with fecal extract supernatants (0.1% w/v) for 24 h in the presence of LPS (1 µg mL^−^^1^) for BV2 cells, LPS (1 µg mL^−^^1^) plus IFN‐γ (10 ng mL^−^^1^) for MA cells, or MPP^+^ (500 µM) for SH‐SY5Y cells.

### 5.17 A. Mucilyticum Culture

*A. mucilyticum* (DSM 112815) was obtained from the BeNa Culture Collection (BNCC364441) and cultured in an anaerobic environment. Fermentation broth was collected by centrifuging at 8000 rpm for 10 min.

### 5.18 Colon Organoids Culture

The isolate of crypts from 6‐week‐old male C57BL/6J mice was performed according to published studies [39]. After the colon was cut open, villi were scraped off, and the remainder was repeatedly cleaned with DPBS to remove the contents and villi. Crypts were released from the colon by incubating on ice for 30 min in DPBS containing 2 mM EDTA, and then filtered through a 70 µm cell strainer, followed by centrifugation at 300 g for 3 min. About 200 crypts were mixed with 50 µL Matrigel (082703, Mogengel Biotech, China) and placed in 24‐well plates. The plate was inverted in an incubator at 37°C for 30 min for the matrix glue to solidify. Then, 500 µL medium (MA‐0817H007, Mogengel Biotech, China) was added. Mature organoids were treated with or without growth medium containing 1 mM MPP^+^, 10% fecal extract or bacterial supernatant for 24 h for immunofluorescence or western blotting.

### 5.19 Immunofluorescence Staining of Organoids

Mature organoids were recovered by organoid recovery solution (MA‐0837DS001P, Mogengel Biotech, China) on ice for 50 min and then fixed in 4% PFA for 30 min. To block non‐specific staining, organoids were incubated with 0.1% Triton X‐100 and 0.2% BSA at 4°C for 15 min. The primary antibodies: rabbit anti‐ZO‐1 (1:1000, 21773‐1‐AP, Proteintech, China), rabbit anti‐Occludin (1:1000, 27260‐1‐AP, Proteintech, China), rabbit anti‐Claudin‐1 (1:200, 28674‐1‐AP, Proteintech, China) were incubated with the organoids at 4°C overnight, and then FITC‐labeled goat anti‐rabbit IgG (1:1000, A0562, Beyotime, China) was used as secondary antibody for incubation at room temperature for 2 h. Organoids were imaged with a laser confocal microscope (Carl Zeiss LSM880, Zeiss, Germany). The mean fluorescence density of mouse colon organoids was analyzed by Image J software.

### 5.20 Statistical Analysis

All data are represented as means ± standard error of mean (SEM), and analyzed with SPSS 22.0 software (IBM SPSS Statistics, Armonk, New York, USA) and GraphPad Prism 8.0 Software (GraphPad Prism Software, Inc., CA, USA). For animal experiments, statistical significance for multiple group comparisons was determined using two‐way ANOVA followed by Tukey's post hoc test. Asterisks (*) indicate significant differences among different treatment groups within the same diet (*p < 0.05, **p < 0.01, ***p < 0.001), while hash symbols (#) indicate significant differences among treatment groups across different diets (#*p* < 0.05, ##*p* < 0.01, ###*p* < 0.001). For cell‐based and colonic organoid experiments, one‐way ANOVA followed by Tukey's post hoc test was used to assess statistical significance, and asterisks (*) indicate significant differences among groups. All graphs were generated using GraphPad Prism 8.0.

## Author Contributions

Y. Shen and T. Li designed the research; T. Li and J. Wu constructed the colonic organoids; T. Li, S. Zhou, L. Zhao, and W. Huang established the MPTP model; S. Zhou and L. Tan operated the behavior experiments; T. Li, M. Li, A. Wang, and Y. Song performed the molecular experiments; T. Li, J. Wu, and W. Huang performed the HPLC experiments; T. Li and M. Li performed the GC‐MS experiments; C. Qiao, W. Zhao, and C. Cui analyzed and interpreted the data; Y. Shen and T. Li wrote the original manuscript; Y. Shen and T. Li revised the manuscript.

## Conflicts of Interest

The authors declare no conflict of interest.

## Supporting information




**Supporting File**: advs74083‐sup‐0001‐SuppMat.docx.

## Data Availability

The data that support the findings of this study are available from the corresponding author upon reasonable request.
