# Supplementary material for: Neuroprotective Effects of Time‐Restricted Feeding Combined With Different Protein Sources in MPTP‐Induced Parkinson's Disease Mice Model and Its Modulatory Impact on Gut Microbiota Metabolism
Source: Adv Sci (Weinh). 2026 Jan 26;13(19):e16502. doi: 10.1002/advs.202516502 (PMC13045433; doi:10.1002/advs.202516502)
Supplement: Supplementary file 1 — Supporting File: advs74083‐sup‐0001‐SuppMat.docx. [file ADVS-13-e16502-s001.docx]

Supporting Information

Neuroprotective Effects of Time-Restricted Feeding Combined with Different Protein Sources in MPTP-Induced Parkinson’s Disease Mice Model and Its Modulatory Impact on Gut Microbiota Metabolism

Ting Li, Jian Wu, Sheng-Yang Zhou, Ming-An Li, Li-Ping Zhao, Ao Wang, Yi-Zhi Song, Wen-Yan Huang, Lu-Lu Tan, Chen-Meng Qiao, Wei-Jiang Zhao, Chun Cui, Yan-Qin Shen*


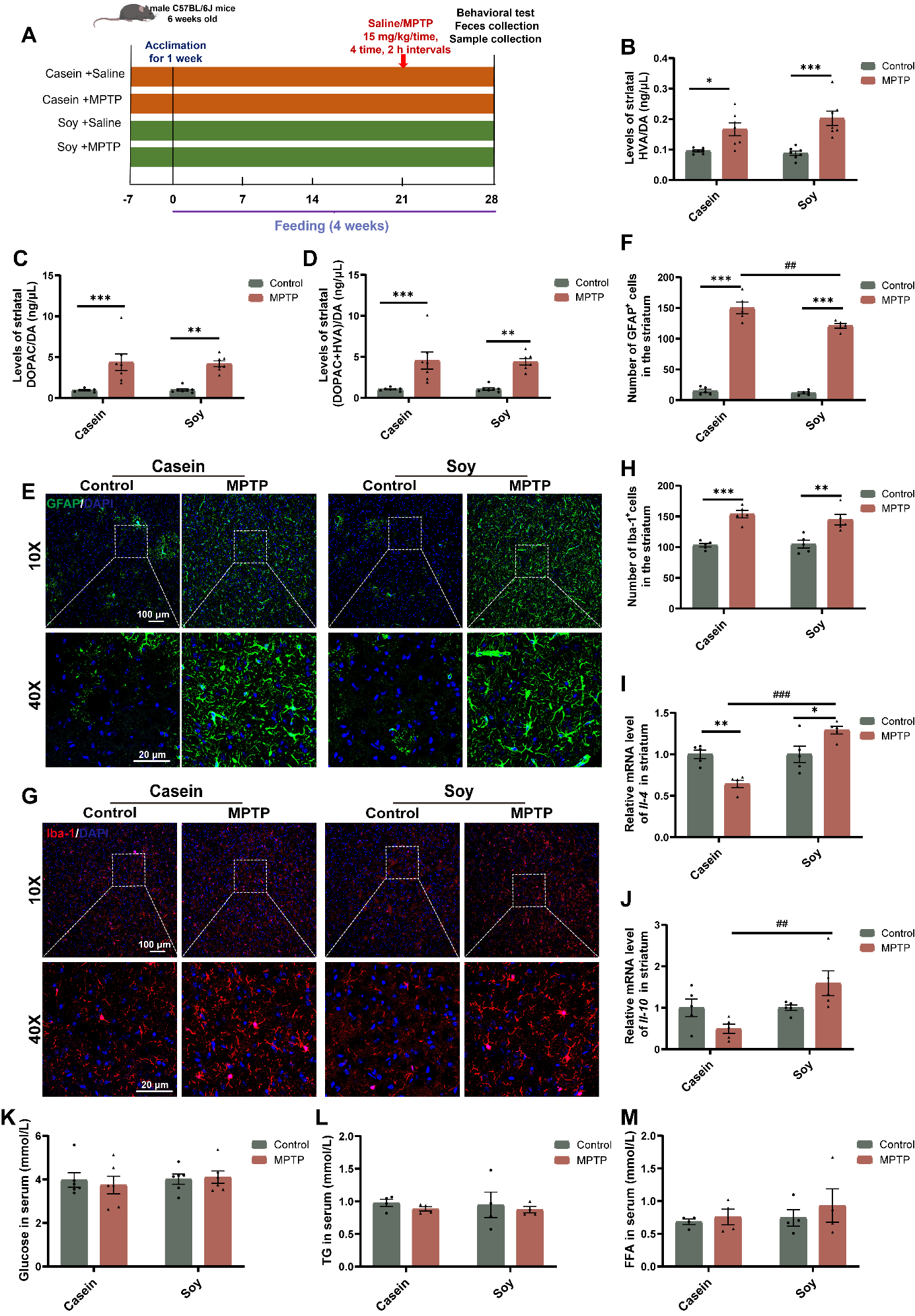


**Supplementary Figure 1** MPTP-induced PD-like pathology depends on the source of dietary protein. A) Timeline for the experimental procedure. B) The ratio of striatal HVA/DA (n = 7). C) The ratio of striatal neurotransmitters DOPAC/DA (n = 7). D) The ratio of striatal neurotransmitters (HVA+DOPAC)/DA (n = 7). E) Representative IF of GFAP (green) in the striatum. Scale bar: 100 μm (10×), 20 μm (40×). F) Quantitative analysis of the number of activated astrocytes in the striatum (n = 5). G) Representative IF of Iba-1 (red) in the striatum. Scale bar: 100 μm (10×), 20 μm (40×). H) Quantitative analysis of the number of activated microglia in the striatum. I) The mRNA level of *Il-4* in the striatum (n = 5). J) The mRNA level of *Il-10* in the striatum (n = 5). K) Serum levels of glucose (n=6). L) Serum levels of triglycerides (n=4). M) Serum levels of free fatty acids (n=4). Error bars represent the mean ± SEM. Statistical significance for multiple group comparisons was determined using two-way ANOVA followed by Tukey’s post hoc test. Asterisks (*) indicate significant differences among different treatment groups within the same diet (*p < 0.05, **p < 0.01, *p < 0.001), while hash symbols (#) indicate significant differences among treatment groups across different diets (#p < 0.05, ##p < 0.01, ###p < 0.001).


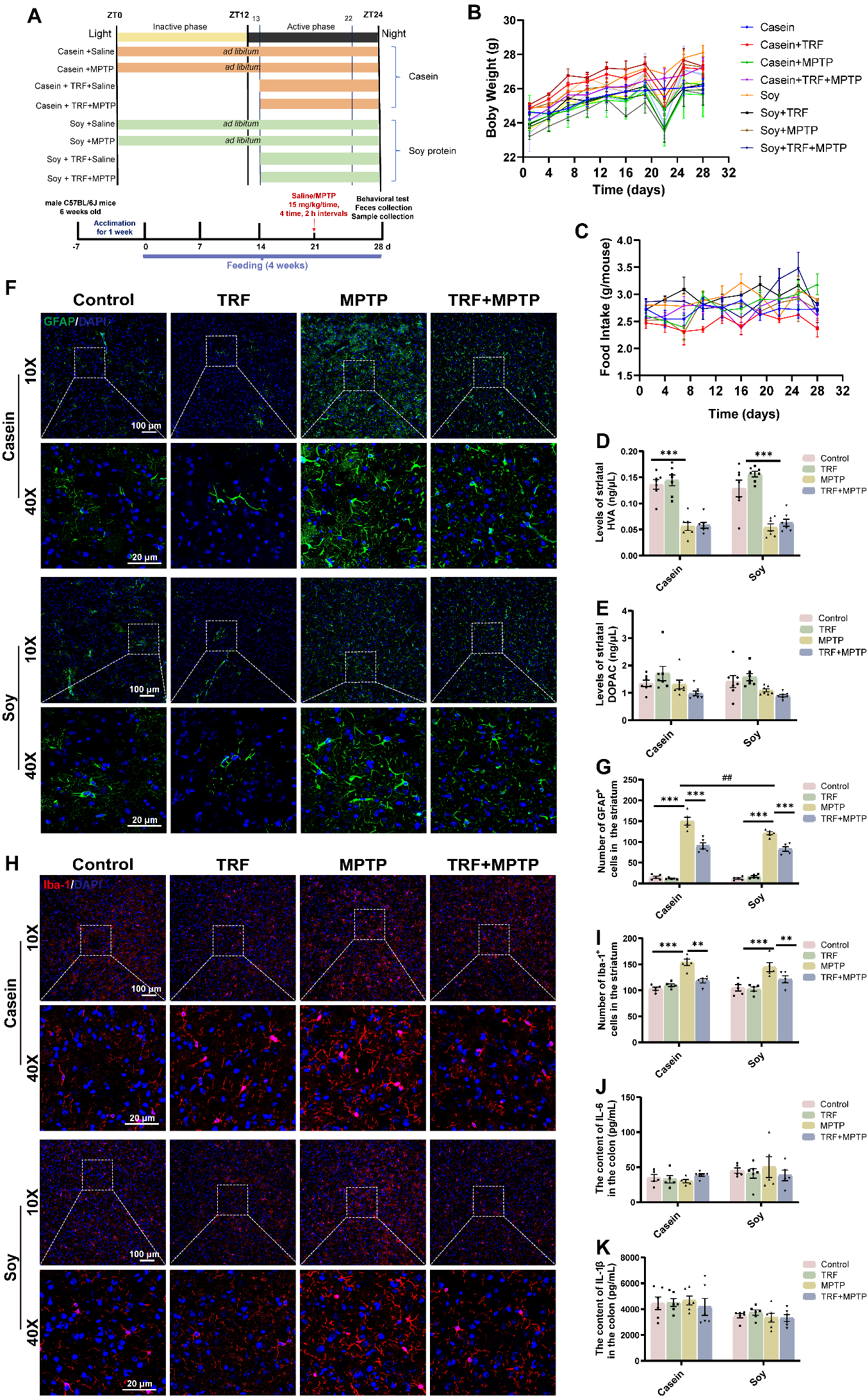


**Supplementary Figure 2** TRF suppresses neuroinflammation in PD mice independently of dietary protein source. A) Timeline for the experimental procedure. B) Body weight changes. C) Average food intake. D) The concentration of striatal metabolites HVA. (n = 7). E) The concentration of striatal metabolites DOPAC (n = 7). F) Representative IF of GFAP (green) in the striatum. Scale bar: 100 μm (10×), 20 μm (40×). G) Quantitative analysis of the number of activated astrocytes in the striatum (n = 5). H) Representative IF of Iba-1 (red) in the striatum. Scale bar: 100 μm (10×), 20 μm (40×). I) Quantitative analysis of the number of activated microglia in the striatum. J) The protein level of IL-6 in the colon (n = 5). K) The protein level of IL-1β in the colon (n = 6). Error bars represent the mean ± SEM. Statistical significance for multiple group comparisons was determined using two-way ANOVA followed by Tukey’s post hoc test. Asterisks (*) indicate significant differences among different treatment groups within the same diet (*p < 0.05, **p < 0.01, *p < 0.001), while hash symbols (#) indicate significant differences among treatment groups across different diets (#p < 0.05, ##p < 0.01, ###p < 0.001).


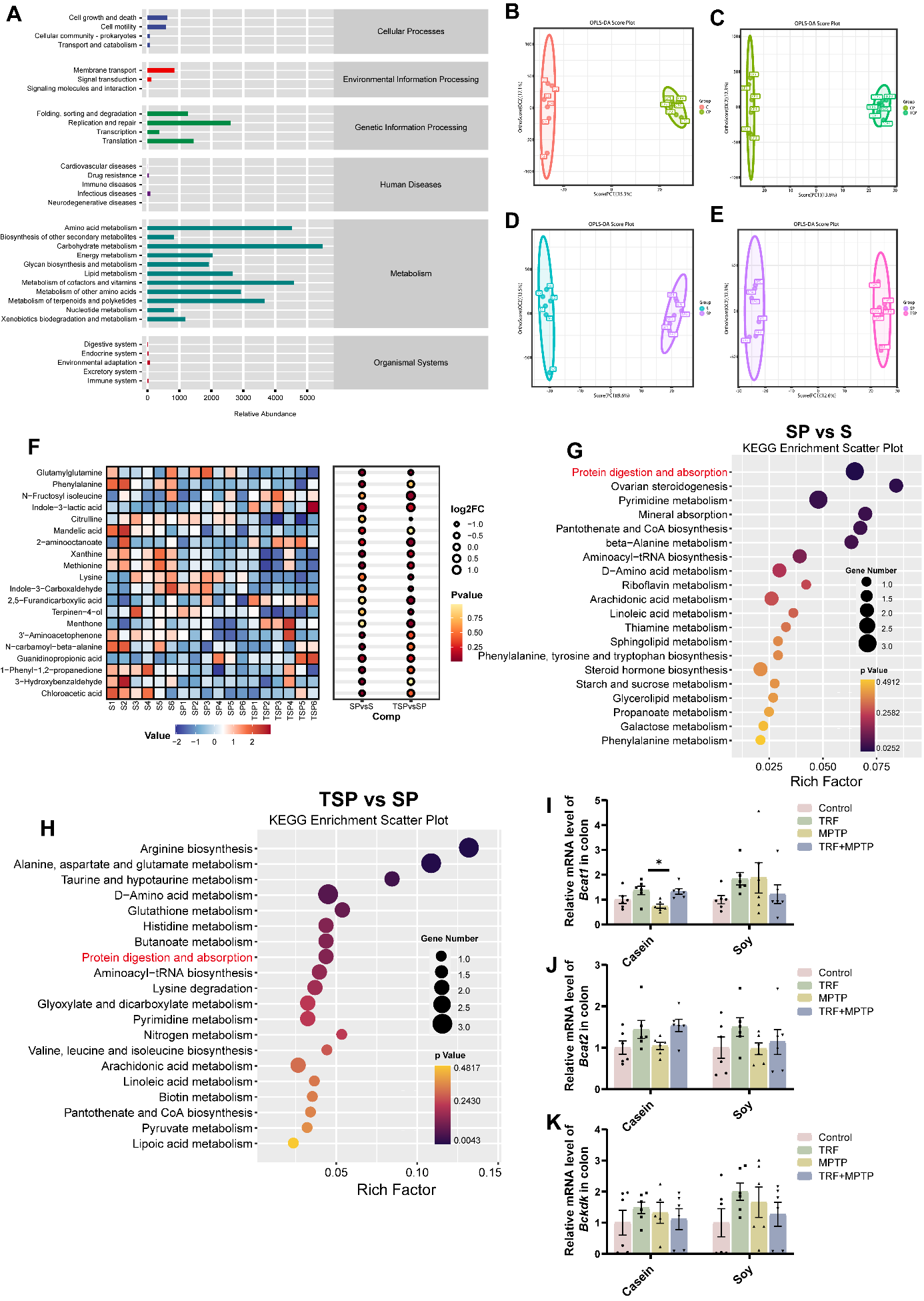


**Supplementary Figure 3** Effects of TRF on BCAAs metabolism. A) The abundance of the differential pathways based on the KEGG. The abscissa is the abundance count of the classification, the ordinate is the functional pathway of KEGG’s second classification level, and the rightmost is the first-level classification to which this pathway belongs. B-E) OPLS-DA score plots comparing metabolomic profiles between different groups (n = 6): CP vs C (B), TCP vs CP (C), SP vs S (D), TSP vs SP (E). F) Heatmap of differential metabolites across groups with log2 fold change (log2FC) and statistical significance (p-value) shown. G-H) KEGG enrichment scatter plots: SP vs S (G), TSP vs SP (H). Circle size represents gene number, and color represents p-value. I-K) Relative mRNA level of *Bcat1*, *Bcat2* and *Bckdk* in the colon (n = 6). C: casein-fed mice, CP: casein-fed PD mice, TCP: TRF-treated casein-fed PD mice; S: soy protein-fed mice, SP: soy protein-fed PD mice, TSP: TRF-treated soy protein-fed PD mice. Error bars represent the mean ± SEM. Statistical significance for multiple group comparisons was determined using two-way ANOVA followed by Tukey’s post hoc test. Asterisks (*) indicate significant differences among different treatment groups within the same diet (*p < 0.05, **p < 0.01, *p < 0.001).


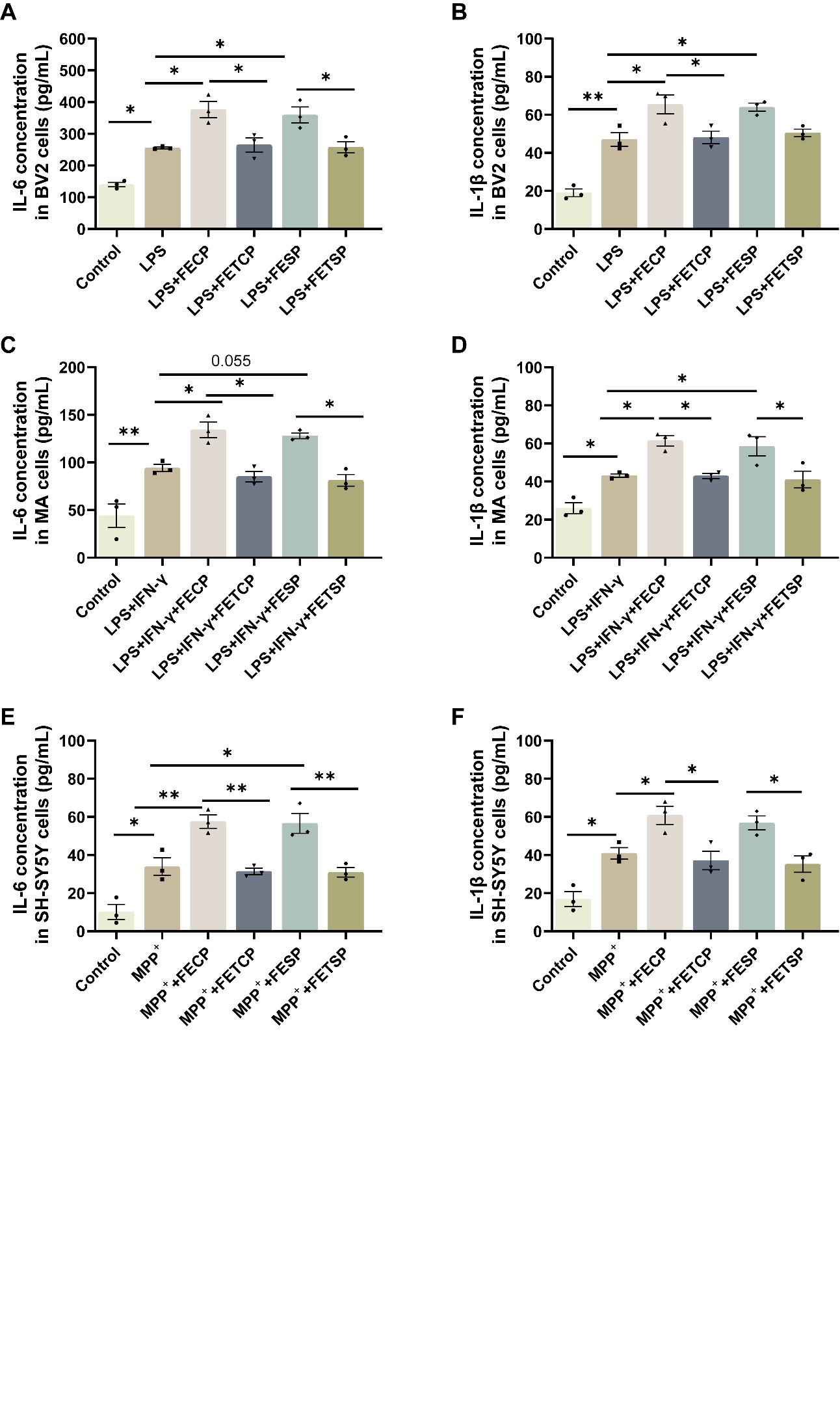


**Supplementary Figure 4** MPTP-induced alterations in gut microbiota impair the neuroinflammation. A) IL-6 concentration in BV2 cells (pg/mL) (n = 3). B) IL-1β concentration in BV2 cells (pg/mL) (n = 3). C) IL-6 concentration in MA cells (pg/mL) (n = 3). D) IL-1β concentration in MA cells (pg/mL) (n = 3). E) IL-6 concentration in SH-SY5Y cells (pg/mL) (n = 3). F) IL-1β concentration in SH-SY5Y cells (pg/mL) (n = 3). FECP: fecal extract from casein-fed PD mice; FETCP: fecal extract from TRF-treated casein-fed PD mice. FESP: fecal extract from soy protein-fed PD mice; FETSP: fecal extract from TRF-treated soy protein -fed PD mice. Error bars represent the mean ± SEM. Statistical significance for multiple group comparisons was determined using one-way ANOVA followed by Tukey’s post hoc test. Asterisks (*) indicate significant differences among different groups (*p < 0.05, **p < 0.01, *p < 0.001).
